# Livestock ownership is associated with higher odds of anaemia among preschool‐aged children, but not women of reproductive age in Ghana

**DOI:** 10.1111/mcn.12604

**Published:** 2018-04-02

**Authors:** Andrew D. Jones, Esi K. Colecraft, Raphael B. Awuah, Sandra Boatemaa, Nathalie J. Lambrecht, Leonard Kofi Adjorlolo, Mark L. Wilson

**Affiliations:** ^1^ Department of Nutritional Sciences, School of Public Health University of Michigan Ann Arbor Michigan USA; ^2^ Nutrition and Food Science Department University of Ghana Accra Ghana; ^3^ Regional Institute for Population Studies University of Ghana Accra Ghana; ^4^ Livestock and Poultry Research Center University of Ghana Accra Ghana; ^5^ Department of Epidemiology, School of Public Health University of Michigan Ann Arbor Michigan USA

**Keywords:** anaemia, animal source foods, Ghana, livestock, malaria, poultry

## Abstract

Livestock ownership may influence anaemia through complex and possibly contradictory mechanisms. In this study, we aimed to determine the association of household livestock ownership with anaemia among women aged 15–49 years and children aged 6–59 months in Ghana and to examine the contribution of animal source foods (ASFs) to consumption patterns as a potential mechanism mediating this association. We analysed data on 4,441 women and 2,735 children from the 2014 Ghana Demographic and Health Survey and 16,772 households from the Ghana Living Standards Survey Round 6. Haemoglobin measurements were used to define anaemia (non‐pregnant women: <120 g/L; children: <110 g/L). Child‐ and household‐level ASF consumption data were collected from 24‐hour food group intake and food consumption and expenditure surveys, respectively. In multiple logistic regression models, household livestock ownership was associated with anaemia among children (OR, 95% CI: 1.5 [1.1, 2.0]), but not women (1.0 [0.83, 1.2]). Household ownership of chickens was associated with higher odds of anaemia among children (1.6 [1.2, 2.2]), but ownership of other animal species was not associated with anaemia among women or children. In path analyses, we observed no evidence of mediation of the association of household livestock ownership with child anaemia by ASF consumption. Ownership of livestock likely has limited importance for consumption of ASFs among young children in Ghana and may in fact place children at an increased risk of anaemia. Further research is needed to elucidate if and how pathogen exposure associated with livestock rearing may underlie this increased risk of anaemia.

**Key messages**

- Household livestock ownership in Ghana, and ownership of poultry in particular, is associated with higher odds of anaemia among young children, but not women.
- Households that own livestock are more likely to consume larger amounts of animal source foods (ASFs), though ASF consumption among young children does not mediate the association of household livestock ownership with anaemia among children.
- Although rearing poultry may facilitate greater household access to eggs and chicken meat, which can contribute to meeting the micronutrient needs of women and children, the potential benefits of these ASFs may be offset by exposure to poultry‐associated pathogens that may increase the risk of anaemia.

## 1 INTRODUCTION

Anaemia among women of reproductive age (WRA) and preschool‐aged children remains an intractable public health problem in many low‐ and middle‐income countries (LMICs), especially in Sub‐Saharan Africa (World Health Organization, 2015). Anaemia is associated with a higher risk of maternal mortality (Ezzati, Lopez, Rodgers, & Murray, 2004), poorer behavioural and developmental outcomes among children (Lozoff et al., 2006; Lozoff, Jimenez, Hagen, Mollen, & Wolf, 2000), and reduced aerobic work capacity and productivity (Horton & Ross, 2003). Though iron deficiency may be responsible for as much as half of all anaemia (Stevens et al., 2013), its contribution is highly variable across geographic and social contexts (Petry et al., 2016), and many other factors influence anaemia risk including parasitic infections, inherited blood disorders, vitamin deficiencies (e.g., folic acid and vitamins B12 and A; Balarajan, Ramakrishnan, Ozaltin, Shankar, & Subramanian, 2011), and acute or chronic immune activation (Weiss & Goodnough, 2005). This complex aetiology suggests the importance of understanding how environmental conditions shape anaemia risk.

Animal husbandry is a ubiquitous practice among rural and peri‐urban households in LMICs and has important potential implications for local environments, household behaviours, and health outcomes (Herrero et al., 2013). Access to meat, milk, and eggs from livestock rearing may increase consumption of anaemia‐mitigating animal source foods (ASFs) either directly through consumption of such foods from own production or indirectly through the purchase of ASFs using earned income from the sale of own‐produced animal products (Hurrell, Reddy, Juillerat, & Cook, 2006; Leroy & Frongillo, 2007; Murphy & Allen, 2003). Yet animal rearing may also exacerbate anaemia risk by (a) increasing exposure to blood‐feeding parasites such as soil‐transmitted helminths (Moore et al., 2015; Pasricha, Drakesmith, Black, Hipgrave, & Biggs, 2013), (b) contributing to non‐specific faecal–oral contamination from animal faeces associated with anaemia of inflammation (Humphrey, 2009), and (c) altering exposure to mosquito vectors of *Plasmodium* parasites by diverting mosquitoes away from human hosts (i.e., zooprophylaxis) or increasing mosquito population density via increased blood meals (i.e., zoopotentiation) (Burkot, Dye, & Graves, 1989; Minakawa, Mutero, Githure, Beier, & Yan, 1999) that can induce malarial anaemia (Haldar & Mohandas, 2009). The few livestock rearing intervention studies that have assessed impacts on anaemia or haemoglobin (Hb) concentrations among women or children were implemented in combination with other interventions, thus obfuscating the independent role of livestock. These interventions, introducing small‐scale, homestead poultry production in concert with promotion of vegetable gardens, field crop production, and/or provision of nutrition education, have shown mixed impacts on anaemia (Galal, Harrison, Abdou, & Zein el Abedin, 1987; Olney, Pedehombga, Ruel, & Dillon, 2015; Olney, Talukder, Iannotti, Ruel, & Quinn, 2009; Osei et al., 2016). The scant observational evidence for the association of livestock rearing with anaemia has similarly shown mixed results with studies reporting higher (Ahenkorah, Nsiah, & Baffoe, 2016; Iannotti et al., 2015) and lower (Flores‐Martinez, Zanello, Shankar, & Poole, 2016; Miller, 2010) odds of anaemia associated with livestock rearing. None of these studies has examined the role of coinfection when assessing associations between livestock and anaemia among children, and no epidemiological studies have addressed differential associations among women and children. Given this limited and inconsistent evidence base, as well as other data indicating a deleterious effect of livestock on child health (Headey et al., 2017; Zambrano, Levy, Menezes, & Freeman, 2014), it is essential to understand how livestock may influence anaemia risk among nutritionally vulnerable populations to help inform interventions that maximize the nutrition and health benefits of livestock, while preventing harm. In this study, we use data from two nationally representative surveys of Ghana to determine the association of household livestock ownership with anaemia among women and children in Ghana and to examine the contribution of ASFs to consumption patterns as a potential mechanism mediating this association.

## 2 SUBJECTS AND METHODS

### 2.1 Data

We analysed data from the 2014 Ghana Demographic and Health Survey (GDHS) and the 2012–2013 Ghana Living Standards Survey Round 6 (GLSS6). The 2014 GDHS was a nationally representative household survey of Ghana that collected data on the fertility, socio‐economic, health, and nutritional status of participating households and individuals using the DHS Phase VI core questionnaire. A two‐stage stratified sampling design was used to select survey enumeration areas (EAs) identified from the 2010 Ghana Population and Housing Census (Ghana Statistical Service, Ghana Health Service, & ICF International, 2015). In the first sampling stage, 427 EAs were selected with probability proportional to size (i.e., number of residential households in the EA) and with independent selection in each of 20 sampling strata. Strata were defined by region and urbanicity. In the second stage of sampling, households were selected using equal probability systematic sampling within each EA. All women aged 15–49 from selected households were eligible to be interviewed, and in half of the selected households, eligible women and children aged 6–59 months were tested for anaemia. Children were also tested for malaria. Field work for the GDHS was carried out from September to December 2014. The GLSS6, a nationally representative survey of Ghana that collected data on household sociodemographic characteristics, agricultural production, consumption, and income, used a similar two‐stage stratified sampling design as the GDHS wherein EAs were selected in a first stage followed by systematic sampling of households within EAs in a second stage (Ghana Statistical Service, 2014). Strata for the GLSS6 were similarly defined based on region and urban location. Fieldwork for the GLSS6 was carried out from October 2012 to October 2013. Our primary analyses were conducted using the GDHS data and complementary analyses of household consumption patterns were carried out using data from the GLSS6.

### 2.2 Measurement of variables

Household livestock ownership was the principal independent variable examined. Data on current ownership of nine different animals or animal groupings were collected in the GDHS (i.e., cattle, horses/donkeys/mules, goats, pigs, rabbits, grasscutters (*Thryonomys swinderianus)*, sheep, chickens, and other poultry). Dichotomous variables indicating any household livestock ownership and ownership of specific animals were used in analyses. Data from the GLSS6 on ownership of any livestock (i.e., cattle, sheep, goats, pigs, rabbits, chicken, guinea fowl (*Numida meleagris*), turkeys, ducks, ostriches, snails, and grasscutters) and participation in wild fish harvesting and aquaculture were also analysed.

Data on the anaemia status of non‐pregnant WRA (15–49 years) and preschool‐aged children (6–59 months) from the GDHS were used as the principal dependent variables, respectively. Capillary blood samples from a finger prick were analysed using HemoCue photometer systems (Hemocue, Inc., Brea, CA) to assess Hb concentrations. Mild, moderate, and severe anaemia, respectively, were defined among non‐pregnant WRA as an Hb concentration of 110–119 g/L, 80–109 g/L, and <80 g/L and among children aged 6–59 months as an Hb concentration of 100–109 g/L, 70–99 g/L, and <70 g/L (World Health Organization, 2011).

We used several household‐level sociodemographic variables from the GDHS in our analyses, as well as data on individual‐level nutrition behaviours and biomarkers of malaria status. These model covariates were selected a priori based on existing theory of determinants of anaemia status in women and children (Bentley & Griffiths, 2003; Tengco, Rayco‐Solon, Solon, Sarol Jr, & Solon, 2008). Sociodemographic variables included the age of women and children, the sex of the head of household and children, the education status of women and mothers of children, the total number of household members, household access to an improved water source (WHO/UNICEF Joint Monitoring Programme for Water Supply and Sanitation) and/or an improved sanitation source (WHO/UNICEF Joint Monitoring Programme for Water Supply and Sanitation), urban residence of the household, and the wealth status of the household assessed using an asset‐based index. Assigned asset weights were generated using a principal components analysis to create standardized asset scores (Rutstein & Johnson, 2004), which were then categorized into quintiles. Additional variables included recent anti‐malaria indoor residual spraying of the household, household access to a mosquito bed net for sleeping, current tobacco use among women (Nordenberg, Yip, & Binkin, 1990), weight and parity of women at the time of the interview, and among children, fever or diarrhoea in the previous 2 weeks, consumption of a high‐dose vitamin A supplement in the previous 6 months, consumption of any iron supplement (i.e., pill, sprinkles with iron, or iron syrup) in the previous 7 days, provision of any drug for intestinal worms in the last 6 months, and breastfeeding status (i.e., ever breastfed and not currently breastfeeding, still breastfeeding, or never breastfed). Thick smear slides of blood from children aged 6–59 months were assessed for malaria parasites via microscopy at the National Public Health Reference Laboratory in Accra. The Noguchi Memorial Institute for Medical Research performed external quality assurance testing for these malaria analyses (Ghana Statistical Service et al., 2015). Data on recent consumption (i.e., previous 24 hr) among children aged 6–59 months of five categories of ASFs were also assessed. These categories included (a) any meat, such as beef, pork, lamb, goat, chicken, or duck; (b) liver, kidney, heart, or other organ meats; (c) fresh or dried fish or shellfish; (d) eggs; and (e) milk, cheese, or other food made from milk. Because these dietary data were only available for a subsample of children from households that were randomly selected for more extensive interviewing, they were not included as covariates in main analyses but were rather examined in separate subanalyses.

Food consumption and expenditure data from the GLSS6 were also analysed. These data were collected during seven repeat visits to households in a 35‐day period. A trained household member recorded daily food consumption and expenditures in a diary that was submitted to a survey enumerator on each visit. In households with no literate members to keep such a diary, an enumerator visited the household daily to record consumption and expenditures (Ghana Statistical Service, 2014). The monetary value of expenditures for 84 purchased food items and of the amount of 62 own‐produced foods consumed during the 35‐day period was collected as part of the diary. These data were aggregated to calculate the total value of household food consumption and expenditures per adult equivalent based on age‐ and sex‐specific energy requirements (FAO, WHO, & UNU, 2004). The following sociodemographic variables were also included in models using GLSS6 data: sex, age, education level, and employment status of household head; household size; urban location; and quintiles of total household expenditure.

### 2.3 Statistical analysis

All analyses were carried out using the Stata statistical software package version 14.2 (2017; StataCorp, College Station, TX, USA). We used the “svyset” command to identify sampling weights, cluster, and strata variables for both the GDHS and GLSS6 data sets. Both data sets required the use of sampling weights to account for the nonproportional allocation of the samples to different regions of the country and to ensure results are representative at the national and domain levels (Ghana Statistical Service et al., 2015). We used the “svy” command in all analyses to estimate Taylor‐linearized standard errors that adjust for the multistage sampling frames of both the GDHS and GLSS6, as well as intra‐household clustering of individuals.

Using GDHS data, means and proportions of key characteristics of households and individuals were calculated separately for samples of women and children. Two‐sided Student's *t* statistics and Pearson's chi‐squared test statistics were also calculated to test for differences in means and proportions, respectively, among households with livestock and with no livestock. Logistic regression was used to model the association of household livestock ownership with anaemia among women and children. Adjusted models controlled for the variables described above as well as regional fixed effects. We also examined separate adjusted models that included the interaction of urban location of household, education status of women and mothers of children, access to an improved water source, and access to an improved sanitation source, respectively, with household livestock ownership. We hypothesized, based on previous evidence, that higher education of women and access to improved water and/or sanitation (linked to urban location) may modify the association between livestock and anaemia by contributing to improved environmental hygiene, animal management practices, and in turn, decreased exposure to harmful pathogens (Prüss‐Ustün et al., 2014; Wagstaff et al., 2004).

We further assessed, using three approaches, the contribution of ASF consumption as a potential mechanism mediating the association of household livestock ownership with anaemia. First, using GDHS data, we examined the association of recent child consumption of ASFs with child anaemia, and the association of household livestock ownership with recent consumption of ASFs using multiple logistic regression models. Second, path analysis was used to examine the potential role of ASF consumption among children in mediating the association of livestock ownership with child anaemia. In separate models, we calculated standardized path coefficients (i.e., regression coefficients converted to standardized Z‐scores) using maximum‐likelihood estimation of direct, indirect, and total effects of household livestock ownership on child anaemia assessing mediation by consumption of any ASFs, meat, dairy, and organ meats, respectively. Robust standard errors were calculated using the Huber–White sandwich estimator. Third, we examined complementary GLSS6 data to understand the contribution of ASFs to household diets in Ghana and to examine the association of household livestock ownership with household food consumption and expenditures on ASFs. Using GLSS6 data, the monetary value per adult equivalent of household food consumption and expenditures was calculated. Differences in the value of consumption and expenditures among households with livestock and with no livestock were tested using the Student's *t* statistic. Multiple linear regression analysis, adjusting for the covariates noted above and regional fixed effects, was used to assess the association of household ownership of livestock with the monetary value per adult equivalent of household food consumption and expenditures.

Associations were considered consistent with random variation at *p* > .05.

### 2.4 Ethical approval

The core data sets of the 2014 GDHS are in the public domain. Access to data for the results of malaria testing among children in the GDHS was obtained with permission from the Survey and Census Directorate of the Ghana Statistical Service. The survey protocol for the 2014 GDHS, including biomarker collection, was reviewed and approved by the Ghana Health Service Ethical Review Committee and the Institutional Review Board of ICF International. The study team was granted access to the GLSS6 data set following a request to the Ghana Statistical Service. Written informed consent of all participants was carried out for both the 2014 GDHS and GLSS6.

## 3 RESULTS

In total, 11,835 households and 9,396 women were successfully interviewed for the 2014 GDHS. Data on anaemia were available for 4,798 of these women from 3,753 households. We excluded 357 pregnant women from analyses yielding a total sample of 4,441 non‐pregnant WRA from 3,480 households. In addition, anaemia data were available for 2,735 children aged 6–59 months from 2,085 households. Dietary data were available for 1,373 of these children. In the GLSS6, 16,772 households were surveyed, all of which were included in analyses using this data set (Table S1).

From the GDHS, 42.3% of women and 65.9% of children were anaemic (Table S2). Among anaemic women, most (78.7%) had mild anaemia, though among anaemic children, moderate anaemia was more prevalent than mild anaemia (56.4% and 40.2% of anaemic children were moderately and mildly anaemic, respectively). Among WRA, two fifths of their households owned livestock (40.5%; Table S2)—a similar percentage to that of households with preschool‐aged children (46.4%). The prevalence of anaemia among women from households that owned livestock was 43.4% as compared with 41.5% among women from households with no livestock. The prevalence of anaemia among children from households that owned livestock was 73.1% as compared to 59.7% among children from households with no livestock.

### 3.1 Household livestock ownership and anaemia

In multiple logistic regression analyses using the GDHS data, and adjusting for household socio‐economic status, demographic characteristics, malaria infection among children, regional fixed effects, and other covariates described above, household livestock ownership was not associated with anaemia among women (OR, 95% CI: 1.0 [0.83, 1.2]) but was associated with anaemia among children (1.5 [1.1, 2.0]; Table 1). This child‐level association persisted after adjusting models for diet diversity among the subsample of children for whom these dietary data were collected (1.6 [1.0, 2.4]; Table S3). In separate models, we observed no statistical interaction of urban location of household, education status of women or mothers of children, or access to an improved water or sanitation source, respectively, with household livestock ownership (Table S4).

**Table 1:** Results of unadjusted and adjusted logistic regression analyses for the association of household livestock ownership with anaemia among non‐pregnant women aged 15–49 years and children aged 6–59 months, respectively

	Women aged 15–49 years	Children aged 6–59 months
	OR [95% CI]	OR [95% CI]
Unadjusted associations		
Household livestock ownership	1.1 [0.93, 1.3]	1.8*** [1.5, 2.3]
Adjusted associations		
Household livestock ownership	1.0 [0.83, 1.2]	1.5** [1.1, 2.0]
Household size	0.99 [0.96, 1.0]	1.0 [0.99, 1.1]
Age, years	0.99* [0.98, 0.99]	.
Age, months	.	0.97*** [0.96, 0.98]
Sex of head of household		
Male (reference)	.	.
Female	0.90 [0.75, 1.1]	1.4* [1.0, 1.9]
Sex of child	.	
Male (reference)	.	.
Female	.	0.94 [0.74, 1.2]
Highest attained education level of woman		
No education (reference)	.	.
Incomplete or complete primary	0.87 [0.69, 1.1]	.
Incomplete or complete secondary	0.81* [0.66, 0.99]	.
Education beyond secondary	0.58** [0.39, 0.87]	.
Highest attained education level of mother		
No education (reference)	.	.
Incomplete or complete primary	.	0.70* [0.50, 0.99]
Incomplete or complete secondary	.	0.60** [0.44, 0.83]
Education beyond secondary	.	0.42* [0.18, 0.99]
Location of household residence		
Urban (reference)	.	.
Rural	0.81* [0.68, 0.96]	0.95 [0.69, 1.3]
Wealth quintiles		
Lowest (reference)	.	.
Low	1.1 [0.86, 1.4]	1.1 [0.77, 1.7]
Middle	0.87 [0.67, 1.1]	0.73 [0.50, 1.1]
High	0.76 [0.57, 1.0]	0.91 [0.58, 1.4)
Highest	0.67* [0.50, 0.91]	0.77 [0.46, 1.3]
Household access to improved water source		
No (reference)	.	.
Yes	1.2* [1.0, 1.4]	1.1 [0.85, 1.5]
Household access to improved sanitation source		
No (reference)	.	.
Yes	1.4** [1.1, 1.8]	0.62* [0.42, 0.90]
Recent anti‐malaria indoor residual spraying of household		
No (reference)	.	.
Yes	0.85 [0.66, 1.1]	0.48** [0.30, 0.78]
Household access to mosquito bed net for sleeping		
No (reference)	.	.
Yes	1.2* [1.0, 1.5]	0.96 [0.69, 1.3]
Current tobacco use		.
No (reference)	.	.
Yes	0.61 [0.09, 4.2]	.
Weight status, kg	0.99** [0.98, 0.99]	.
Parity	1.0 [0.98, 1.0]	.
Fever in previous 2 weeks		
No (reference)	.	.
Yes	.	2.1*** [1.5, 2.9]
Diarrhoea in previous 2 weeks		
No (reference)	.	.
Yes	.	1.1 [0.77, 1.6]
Consumption of vitamin A supplement in previous 6 months		
No (reference)	.	.
Yes	.	0.98 [0.75, 1.3]
Consumption of iron supplement in previous 7 days		
No (reference)	.	.
Yes	.	1.1 [0.77, 1.4]
Treatment for intestinal worms in the last 6 months		
No (reference)	.	.
Yes	.	0.98 [0.76, 1.3]
Breastfeeding status		
Never breastfed (reference)	.	.
Ever breastfed and not currently breastfeeding	.	1.0 [0.39, 2.6]
Still breastfeeding	.	1.2 [0.44, 3.3]
Presence of malaria parasites		
No (reference)	.	.
Yes	.	3.0*** [2.1, 4.2]

*Note*. Values are odds ratios (95% CI) from logistic regression models of the association of household livestock ownership with anaemia status. Adjusted models are multiple logistic regression models that control for all covariates shown as well as regional fixed effects. Standard errors are adjusted for intra‐household clustering and the multistage sampling frame of the 2014 Ghana Demographic and Health Survey through the use of Taylor‐linearized standard errors. Anaemia is modelled as a dichotomous variable defined among women as haemoglobin <120 g/L, and among children as haemoglobin <110 g/L. Sample sizes: unadjusted model for women: *n* = 4,441; adjusted model for women: n = 4,392; unadjusted model for children: *n* = 2,735; adjusted model for children: n = 2,336.

* *p* < .05

** *p* < .01

*** *p* < .001.

In subanalyses, again using multiple logistic regression models with GDHS data, the association of household ownership of specific livestock species with anaemia among women and children, respectively, was assessed. Only chicken ownership was associated with higher odds of anaemia, and only among children, both from households owning chickens and other livestock (1.6 [1.2, 2.2]) and from households owning chickens and no other livestock (1.9 [1.3, 2.6]; Table 2).

**Table 2:** Results of multiple logistic regression analyses for the association of household ownership of specific livestock species with anaemia among women aged 15–49 years and children aged 6–59 months, respectively

	Women aged 15–49 years		Children aged 6–59 months	
	*n*	OR [95% CI]	*n*	OR [95% CI]
Household livestock ownership				
Non‐dairy cattle	4,392	1.3 [0.92, 1.9]	2,336	1.2 [0.70, 1.9]
Dairy cattle	4,392	0.77 [0.51, 1.2]	2,336	1.0 [0.58, 1.8]
Sheep	4,392	1.0 [0.77, 1.4]	2,336	0.92 [0.67, 1.3]
Goats	4,392	0.91 [0.75, 1.1]	2,336	1.1 [0.78, 1.5]
Pigs	4,392	0.77 [0.55, 1.1]	2,336	0.90 [0.48, 1.7]
Chickens	4,390	0.95 [0.79, 1.1]	2,336	1.6** [1.2, 2.2]
Chickens (and no other livestock)	4,392	1.1 [0.89, 1.3]	2,336	1.9*** [1.3, 2.6]

*Note*. Values are odds ratios (95% CI) from multiple logistic regression models of the association of household ownership of specific livestock species with anaemia status. Full model results with estimates for select covariates are presented in Tables S5 and S6. Standard errors are adjusted for intra‐household clustering and the multistage sampling frame of the 2014 Ghana Demographic and Health Survey through the use of Taylor‐linearized standard errors. Anaemia is modelled as a dichotomous variable defined among women as haemoglobin <120 g/L, and among children as haemoglobin <110 g/L.

* *p* < .05

** *p* < .01

*** *p* < .001.

### 3.2 Consumption of ASFs and associations with livestock ownership and anaemia

Examining the GLSS6 data, ASFs contributed more than a quarter (28.3%) of the value of all household food consumption and expenditures, and 10% of the value of food consumed from own production (Tables 3 and S5). Fish accounted for most of the value of total household consumption and expenditure on ASFs (58.4%), whereas chicken meat and eggs accounted for 11.8% and 4.9%, respectively. Complementary descriptive data from the GDHS indicated that 39.8% of children aged 6–59 months consumed fish in the past 24 hr, whereas 11.4% consumed any meat (i.e., beef, pork, lamb, goat, chicken, or duck), 16.7% consumed eggs, and 2.1% consumed dairy (Table S2). From the GLSS6, the value of household consumption and expenditures for all foods, all ASFs, and all categories of ASFs (except chicken) was higher among households that did not own livestock as compared to households with livestock (*p* < .05; Table 3). Similarly, the value of expenditures on all purchased food, ASFs, and all categories of ASFs was also higher among households that did not own livestock as compared to households with livestock (*p* < .05; Table S5).

**Table 3:** Monetary value per adult equivalent of household food consumption and expenditures in the previous 35 days among participants of the Ghana Living Standards Survey Round 6

	Total sample		Households with livestock		Households with no livestock		
	*n*	Mean [95% CI]	*n*	Mean [95% CI]	*n*	Mean [95% CI]	*t* statistic
Value of household food consumption (own produced food) and expenditures (purchased food)							
All food consumption per adult equivalent	16,772	150 [145, 155]	7,273	136 [129, 143]	9,499	157 [150, 163]	7.5***
ASF consumption per adult equivalent	16,766	42.5 [40.7, 44.2]	7,273	34.4 [32.1, 36.6]	9,493	46.6 [44.4, 48.8]	16.2***
Meat consumption per adult equivalent	16,766	7.3 [6.7, 8.0]	7,273	6.6 [5.1, 8.1]	9,493	7.7 [7.1, 8.3]	2.9**
Chicken consumption per adult equivalent	16,766	5.0 [4.6, 5.3]	7,273	5.3 [4.8, 5.8]	9,493	4.8 [4.4, 5.2]	−3.1**
Fish consumption per adult equivalent	16,766	24.8 [23.6, 26.0]	7,273	19.4 [18.1, 20.8]	9,493	27.5 [26.0, 28.9]	18.3***
Milk consumption per adult equivalent	16,766	3.1 [2.9, 3.3]	7,273	1.4 [1.3, 1.5]	9,493	3.9 [3.7, 4.2]	28.8***
Egg consumption per adult equivalent	16,766	2.1 [1.9, 2.2]	7,273	1.1 [1.0, 1.2]	9,493	2.5 [2.3, 2.7]	16.1***

*Note*. Values are means (95% CI) in units of Ghanaian cedi adjusted for the multistage sampling frame of the Ghana Living Standards Survey Round 6 through the use of Taylor‐linearized standard errors. Sample sizes shown are nominal sample sizes. The two‐sided Student's t‐statistics shown test for differences in means for characteristics among households with livestock and with no livestock. Own produced “meat” includes beef, mutton, pork, goat, other domestic meat, and wild game; purchased “meat” includes corned beef, pork, beef, goat, mutton, bushmeat or wild game, and other meat; purchased “fish” includes crustaceans, fish (fresh, frozen, dried, canned, fried, smoked, salted); purchased “milk” includes fresh, powdered, or tinned milk. ASF = animal source foods.

* *p* < .05

** *p* < .01

*** *p* < .001

In multiple linear regression models using GLSS6 data, livestock ownership was associated with a higher value of household food consumption and expenditures per adult equivalent of all food (partial regression coefficient (95% CI): 26.1 [17.9, 34.3]); ASFs (6.8 [4.1, 9.6]); chicken meat (2.0 [1.4, 2.6]); and fish (3.2 [1.8, 4.6]; Tables 4 and S6). Ownership of chickens was associated with a higher value of household food consumption and expenditures per adult equivalent of chicken meat (2.6 [1.9, 3.2]) and eggs (0.14 [0.00, 0.28]). Similarly, ownership of chickens was associated with a higher value of household food consumption from own production per adult equivalent of chicken meat (3.2 [2.7, 3.7]) and eggs (0.46 [0.37, 0.55]; Table S6). Using data from the 2014 GDHS, in multiple logistic regression models, the associations of household ownership of any livestock or specific animals (i.e., cattle, sheep, goats, pigs, and chicken) with recent consumption of different ASFs (i.e., meat, fish, eggs, dairy, and organ meats) by children aged 6–59 months were all consistent with random variation.

**Table 4:** Results of multiple linear regression analyses for the association of household ownership of livestock with the monetary value per adult equivalent of household food consumption and expenditures in the previous 35 days, by food type, among participants of the Ghana Living Standards Survey Round 6

	Value of household food consumption (own produced food) and expenditures (purchased food) per adult equivalent						
	All food Coefficient [95% CI]	ASFs Coefficient [95% CI]	Meat Coefficient [95% CI]	Chicken Coefficient [95% CI]	Fish Coefficient [95% CI]	Milk Coefficient [95% CI]	Eggs Coefficient [95% CI]
Household ownership of any livestock	26.1*** [17.9, 34.3]	6.8*** [4.1, 9.6]	1.7 [−0.15, 3.5]	2.0*** [1.4, 2.6]	3.2*** [1.8, 4.6]	−0.28** [−0.47, −0.10]	0.03 [−0.12, 0.18]
Household ownership of cattle	.	.	4.9* [0.52, 9.2]	.	.	0.30 [−0.06, 0.65]	.
Household ownership of sheep	.	.	3.3 [−0.29, 6.9]	.	.	.	.
Household ownership of goats	.	.	1.6 [−0.38, 3.6]	.	.	.	.
Household ownership of pigs	.	.	5.8 [−0.49, 12.1]	.	.	.	.
Household ownership of chickens	.	.	.	2.6*** [1.9, 3.2]	.	.	0.14* [0.00, 0.28]
Household participation in wild fish capture	.	.	.	.	21.9* [1.6, 42.3]	.	.
Household participation in fish farming	.	.	.	.	6.4 [−1.6, 14.4]	.	.

*Note*. Values are partial regression coefficients (95% CI) from multiple linear regression models of the association of household ownership of livestock with the monetary value per adult equivalent of household food consumption and expenditures, by food type. Models are adjusted for the sex, age, education level, and employment status of household head, household size, urban location, quintiles of total household expenditure, and regional fixed effects. Full model results with estimates for select covariates in select models are presented in Table S3. Standard errors are adjusted for the multistage sampling frame of the Ghana Living Standards Survey Round 6 through the use of Taylor‐linearized standard errors. Monetary value data are in units of Ghanaian cedi. Sample size: *n* = 16,246. Animal source foods include all categories of such foods shown; own produced “meat” includes beef, mutton, pork, goat, other domestic meat, and wild game; purchased “meat” includes corned beef, pork, beef, goat, mutton, bushmeat or wild game, and other meat; purchased “fish” includes crustaceans, fish (fresh, frozen, dried, canned, fried, smoked, salted); purchased “milk” includes fresh, powdered, or tinned milk. ASF = animal source foods.

* *p* < .05

** *p* < .01

*** *p* < .001

Using GDHS data, children who recently consumed organ meat had lower odds of anaemia (0.43 [0.19, 0.99]), whereas recent consumption of dairy was marginally associated with higher odds of anaemia (2.1 [0.95, 4.8] [*p* = .07]). The associations of recent consumption of all other ASFs with anaemia among children were consistent with random variation. In path analyses, we similarly observed no evidence of mediation of the association of livestock ownership with child anaemia by consumption of ASFs (Figure S1; Table S7). The indirect effect of household livestock ownership on child anaemia as mediated by consumption of ASFs was consistent with random variation in models examining children's recent consumption of any ASFs, meat, dairy, and organ meats, respectively.

## 4 DISCUSSION

Independent of socio‐economic status and infection indicators, livestock ownership in Ghana was associated with higher odds of anaemia among children aged 6–59 months but not among WRA. In particular, household ownership of chickens was associated with higher odds of anaemia among children. Ownership of no other animal species was independently associated with anaemia among either women or children. Except for fish, all other ASF types individually contributed <5% of the value of total household food consumption and expenditures, and all own produced ASFs contributed <2% of this value. Children from households that owned livestock were not more likely to have recently consumed ASFs. Furthermore, recent consumption of ASFs was not associated with lower odds of anaemia among children except for consumption of organ meat, and we observed no evidence of mediation of the association of household livestock ownership with child anaemia by ASF consumption. Therefore, although livestock ownership was associated with a higher value of household ASF consumption and expenditures, livestock ownership may have limited importance for ASF consumption among young children in Ghana and is most strongly associated with anaemia through nonnutritional pathways.

Livestock serve many different functions among households in LMICs (Randolph et al., 2007); use of animals for direct consumption is often a low priority use. Some evidence suggests that livestock ownership translates into increased consumption of dairy and meat (Azzarri, Zezza, Haile, & Cross, 2015; Rawlins, Pimkina, Barrett, Pedersen, & Wydick, 2014). However, keeping livestock or enhancing livestock productivity does not guarantee increased consumption of ASFs, particularly if the livestock are predominantly sold for income (Hoffman, Riethmuller, & Steane, 2003). Furthermore, ASFs vary with respect to their potential to mitigate anaemia. Organ meat and whole fish are rich sources of bioavailable iron and vitamin A which can mitigate anaemia (Neumann, Harris, & Rogers, 2002), though there is little evidence of this potential for eggs and unfortified dairy (Dror & Allen, 2011; Shapira, 2009). At the same time, eggs and dairy are the ASFs most likely to be produced for own consumption among households that keep livestock (Wilson et al., 2005). If not iron‐fortified (Stekel et al., 1988; Villalpando, Shamah, Rivera, Lara, & Monterrubio, 2006), cow's milk may actually increase anaemia (Oliveira & Osório, 2005; Ziegler, 2011). Indeed, recent consumption of dairy by Ghanaian children in this study showed a trend consistent with higher odds of anaemia. Though plausible that differences in ethnicity or cultural traditions that are also associated with food access and distinct feeding behaviours may underlie this observed trend, we found that neither ethnic group nor region were associated with dairy consumption in this study (data not shown). However, we cannot rule out reverse causality as an explanation for the observed trend between dairy consumption and anaemia among children (i.e., caregivers may preferentially feed cow's milk or other dairy foods to undernourished, anaemic children; Marquis, Habicht, Lanata, Black, & Rasmussen, 1997).

Household ownership of chickens, but no other livestock type, was independently associated with higher odds of anaemia among young children. The magnitude of this association was even stronger among households that owned no other livestock but chickens. The small number of households that owned dairy cattle and pigs limited the statistical power available to assess associations among these two animal groupings. However, this is not a likely explanation for the associations examined with small ruminants (i.e., nearly one third and one half of households owned sheep and goats, respectively) and does not explain the statistically significant associations observed for poultry ownership. Evidence from other settings indicates that corralling poultry inside human dwellings is associated with lower child height‐for‐age Z‐scores (George et al., 2015b; Headey & Hirvonen, 2016; Weisz et al., 2012). Young children in close contact with chickens are known to handle or even consume chicken faeces and contaminated soil, which may increase infections of pathogenic bacteria (e.g., *Campylobacter jejuni*, *Escherichia coli*; George et al., 2015a; Marquis et al., 1990; Ngure et al., 2013). Such infections are a major cause of childhood diarrhoea in LMICs (Guerrant, Hughes, Lima, & Crane, 1990). Asymptomatic infections may also impair child growth (Kotloff et al., 2013; Lee et al., 2013), possibly due to changes in intestinal structure and barrier function brought about by repeated exposure to enteric pathogens (Campbell, Elia, & Lunn, 2003; Lin et al., 2013). The same inflammation‐mediated mechanisms via which this gut disorder—termed environmental enteric dysfunction (EED)—is thought to stunt linear growth (Humphrey, 2009; Prendergast et al., 2014) could be integral to its role in the aetiology of anaemia in children (Morceau, Dicato, & Diederich, 2009; Prendergast et al., 2015). If children are especially susceptible to the pathogenic bacteria associated with livestock production (Osbjer et al., 2016; Vasco, Graham, & Trueba, 2016), this may explain why children, but not women, from livestock owning households were more likely to be anaemic.

The observed association of livestock ownership with child anaemia was independent of infection with malaria parasites. Much previous evidence suggests that malaria is an important determinant of anaemia among children in this population (Ehrhardt et al., 2006; Korenromp, Armstrong‐Schellenberg, Williams, Nahlen, & Snow, 2004; Wirth et al., 2016). *Plasmodium* parasites simultaneously consume haem, remove circulating erythrocytes, and decrease production of erythrocytes in bone marrow, thus inducing or exacerbating anaemia (Haldar & Mohandas, 2009). However, the extent to which animal husbandry may alter malaria risk is not clear, and both zooprophylactic and zoopotentiating effects have been observed depending on the preference of local mosquito species for human hosts, livestock distance from human sleeping quarters, and the use of protective measures (Asale, Duchateau, Devleesschauwer, Huisman, & Yewhalaw, 2017; Donnelly, Berrang‐Ford, Ross, & Michel, 2015). Most studies assessing the relationship between livestock keeping and malaria have examined the rearing of cattle and small ruminants with almost no evidence supporting a biological mechanism linking exposure to chickens and risk of malaria (Donnelly et al., 2015; Jaleta, Hill, Birgersson, Tekie, & Ignell, 2016). Therefore, malaria was not hypothesized to be on the causal pathway between chicken ownership and anaemia. Rather, we adjusted for it in regression models as a confounder because it is a known independent determinant of anaemia among children in this population. Given that the association between household livestock ownership and child anaemia was observed independent of the malaria status of children, and that no evidence was observed for mediation of this association by diet, it is plausible that other infections, possibly asymptomatic, related to livestock keeping and poultry rearing in particular, may in part underlie the observed results.

Although this study used large, nationally representative data sets and adjusted for many potentially confounding covariates, it had several limitations. First, the data were observational and collected at a single time point. Therefore, though multiple regression models were used to control for confounding bias, we cannot strictly determine the causal nature or temporal patterns of the observed associations. Second, dietary data in the 2014 GDHS were limited to a qualitative assessment of food group diversity among a subsample of children. These data were not strong predictors of anaemia status, and including dietary variables in regressions did not alter the observed associations of livestock and anaemia. However, it is possible that quantitative diet data for all children would have yielded different results, particularly if the amount of ASF consumed, and not simply the presence of ASFs in diet, is an important determinant of anaemia status among children. Indeed, ASFs included in family meals in Ghana are frequently consumed by children only in small amounts (Colecraft et al., 2006). Furthermore, dietary data based on a single 24‐hr recall period are not necessarily representative of a child's usual diet. In addition, child diets and caregiver feeding practices differ for infants as compared with children nearing school age. These differences could alter the magnitude of or mechanisms underlying the association of household livestock ownership with anaemia. However, we did not find any evidence of child age acting as an effect modifier of this association (data not shown), and therefore analyses were presented in aggregate for all preschool‐aged children. Third, it is possible that the observed association between livestock ownership and anaemia is confounded by household wealth. The proportion of poultry‐owning households increased in a linear fashion with decreasing wealth quintile among both the samples of women and children in the 2014 GDHS (data not shown). Yet the observed association of livestock ownership and anaemia was independent of household wealth, and when examining this association stratified by wealth quintiles, no differences across quintiles were observed (data not shown). The contrasting results for the associations of livestock and anaemia among women and children also suggest that wealth may not be responsible for the observed associations given that women and children are both vulnerable to the deleterious effects of poverty. Nonetheless, residual confounding by household economic status cannot be ruled out in these analyses. Fourth, although our models controlled for recent treatment for intestinal worms, no data were available in the 2014 GDHS for current helminth infection in children at the time of the survey, nor were data available to assess iron deficiency in children or women, nor to consider genetic blood disorders such as Thalassemia—a potentially important causal factor for anaemia in West African populations (Weatherall, 2001). We also had no information from the available data sets on where animals were housed and therefore could not assess differences in anaemia among women and children from households that corral animals inside living quarters compared with those that do not. Fifth, the GLSS6 only provided data on the value of household consumption and expenditures, and therefore, individual food intake data could not be analysed. Nonetheless, though data from consumption and expenditures surveys are not a direct assessment of individual dietary intake, such data do produce nutrient intake estimates that are strongly associated with nutrient intake estimates from dietary intake surveys (Jariseta, Dary, Fiedler, & Franklin, 2012; Naska, Vasdekis, & Trichopoulou, 2001). Furthermore, analysing these data on a per adult equivalent basis accounts for intra‐household differences in energy and nutrient needs based on household composition and allows for more consistent comparisons across households (Claro, Levy, Bandoni, & Mondini, 2010). Sixth, assessing livestock ownership in a binary fashion could mask heterogeneity important for understanding the association of livestock ownership with anaemia. In addition to the analyses presented in this study, we also evaluated livestock as a continuous count variable and as livestock units based on animal body weight (Jahnke, 1982). In both cases, we observed similar though somewhat attenuated magnitudes of association between livestock ownership and anaemia as those using the binary livestock ownership variable (data not shown). Including squared terms for these continuous livestock variables in models provided no evidence of nonlinearity in the association of livestock ownership and anaemia. Given these results, the binary livestock ownership variable assessed is likely appropriate for representing these relationships and also aligns with the specification of variables in other similar studies (Flores‐Martinez et al., 2016; Iannotti et al., 2015). Finally, though we analysed anaemia among women and children in separate models, it is possible to treat these dependent variables as correlated responses and analyse them using bivariate mixed models. This approach could be explored in future research.

## 5 CONCLUSION

Progress in reducing anaemia in Ghana, as in most LMICs, has been slow, and large numbers of women and children continue to suffer from the disease (i.e., 45% and 42% of WRA, and 76% and 66% of children aged 6–59 months in Ghana were anaemic in 2003 and 2014, respectively [Ghana Statistical Service et al., 2015]). Novel interventions that address the underlying environmental determinants of anaemia are needed to complement existing approaches that largely centre on addressing proximal causes of the problem (i.e., control of malaria and helminth infections, and nutritional deficiencies; SPRING & Ghana Health Service, 2016). Exposure to livestock may be one such environmental determinant that is especially important for influencing anaemia risk among children; however, the mechanisms underlying this association are complex, possibly contradictory, and require further clarification. Improved hygiene and sanitation practices can positively impact child health (Curtis & Cairncross, 2003; Esrey, 1996), though emerging evidence suggests that improving such practices alone may not be sufficient to resolve poor health outcomes among children (Luby, 2017). Reducing contact with chicken faeces may be especially important, though the barriers to doing so could be substantial. Corralling chickens, for example, can incur added costs for feed and coop materials, and/or additional labour burdens—all of which may be difficult for low‐income families to bear (Harvey et al., 2003; Martinez et al., 2013). Corralling chickens may also concentrate pathogens, thus leading to greater infection (Oberhelman et al., 2006). Furthermore, if infection risk is predominantly driven by contact with neighbouring animals, local corralling may not address the problem (Oberhelman et al., 2003). Research is needed that jointly assesses the diverse direct and indirect pathways linking livestock and anaemia (e.g., diet; exposure to animal faeces, animal slaughter waste [Osbjer et al., 2016], and zoonotic pathogens linked to livestock disease [Mosites et al., 2016]); altering human vector interactions [Donnelly et al., 2015]). Understanding the relative importance of these factors across contexts and their relationships will help to elucidate the complex aetiology of anaemia among agricultural households and inform solutions that maximize the potential dietary benefits of animal production while minimizing disease risks.

## CONFLICTS OF INTEREST

The authors declare that they have no conflicts of interest.

## CONTRIBUTIONS

ADJ designed the research, performed statistical analyses, and wrote the first draft of the manuscript; RBA and SB compiled and cleaned the initial dataset; all authors contributed to the writing and revision of the manuscript and read and approved the final manuscript.

## Supporting information


**Figure S1.** Path diagram of the direct and indirect effects of household livestock ownership on child anemia with mediation by consumption of any animal‐source foods.
**Table S1.** Sociodemographic characteristics of households participating in the Ghana Living Standards Survey Round 6, by household livestock ownership.
**Table S2.** Sociodemographic characteristics, and health and nutrition indicators among non‐pregnant women aged 15–49 years and children aged 6–59 months from the 2014 Ghana Demographic and Health Survey, by household livestock ownership.
**Table S3**. Results of multiple logistic regression analyses for the association of household livestock ownership with anemia among children aged 6–59 months using the sub‐sample of children for which dietary data were available in the 2014 Ghana Demographic and Health Survey.
**Table S4**. Results of interaction models of logistic regression analyses examining the association of household livestock ownership with anemia among non‐pregnant women aged 15–49 years and children aged 6–59 months, respectively.
**Table S5**. Monetary value per adult equivalent of household food consumption and expenditures in the previous 35 days among participants of the Ghana Living Standards Survey Round 6, by own produced food and purchased food.
**Table S6**. Covariate results from multiple linear regression analyses for the association of household ownership of livestock with the monetary value per adult equivalent of household food consumption and expenditures in the previous 35 days, by food type, among participants of the Ghana Living Standards Survey Round 6.
**Table S7**. Direct, indirect, and total effects of household livestock ownership on child anemia assessing mediation by recent consumption of animal‐source foods.
