# Supplementary material for: Livestock ownership is associated with higher odds of anaemia among preschool‐aged children, but not women of reproductive age in Ghana
Source: Matern Child Nutr. 2018 Apr 2;14(3):e12604. doi: 10.1111/mcn.12604 (PMC6055803; doi:10.1111/mcn.12604)
Supplement: Supplementary file 1 — Figure S1. Path diagram of the direct and indirect effects of household livestock ownership on child anemia with mediation by consumption of any animal‐source foods. Table S1. Sociodemographic characteristics of households participating in the Ghana Living Standards Survey Round 6, by household livestock ownership. Table S2. Sociodemographic characteristics, and health and nutrition indicators among non‐pregnant women aged 15–49 years and children aged 6–59 months from the 2014 Ghana Demographic and Health Survey, by household livestock ownership. Table S3. Results of multiple logistic regression analyses for the association of household livestock ownership with anemia among children aged 6–59 months using the sub‐sample of children for which dietary data were available in the 2014 Ghana Demographic and Health Survey. Table S4. Results of interaction models of logistic regression analyses examining the association of household livestock ownership with anemia among non‐pregnant women aged 15–49 years and children aged 6–59 months, respectively. Table S5. Monetary value per adult equivalent of household food consumption and expenditures in the previous 35 days among participants of the Ghana Living Standards Survey Round 6, by own produced food and purchased food. Table S6. Covariate results from multiple linear regression analyses for the association of household ownership of livestock with the monetary value per adult equivalent of household food consumption and expenditures in the previous 35 days, by food type, among participants of the Ghana Living Standards Survey Round 6. Table S7. Direct, indirect, and total effects of household livestock ownership on child anemia assessing mediation by recent consumption of animal‐source foods. [file MCN-14-e12604-s001.docx › MCN_12604-sup-0001-OnlineSupplementalMaterial_revised.docx]

Online Supplemental Material

**Supplemental Figure 1.** Path diagram of the direct and indirect effects of household livestock ownership on child anemia with mediation by consumption of any animal-source foods.

^a^Coefficients are standardized path coefficients from models using maximum-likelihood estimation with robust standard errors; ^b^Abbreviations: ASFs: animal-source foods.

**Supplemental Table 1.** Sociodemographic characteristics of households participating in the Ghana Living Standards Survey Round 6, by household livestock ownership.

|  | Households with any livestock | | Households with no livestock | |  |
| --- | --- | --- | --- | --- | --- |
|  | n | % or mean  (95% CI) | n | % or mean  (95% CI) | t-statistic or Χ^2^ |
| Number of households | 7,273 |  | 9,499 |  |  |
|  |  |  |  |  |  |
| Household size | 7,273 | 5.2 (5.1, 5.3) | 9,499 | 3.4 (3.3, 3.5) | -48.9*** |
| Age of head of household, years | 7,273 | 48.5 (48.0, 49.0) | 9,499 | 43.3 (42.9, 43.8) | -19.6*** |
| Sex of head of household, % | 7,273 |  | 9,499 |  | 616*** |
| Female |  | 20.2 (18.8, 21.7) |  | 35.7 (34.2, 37.3) |  |
| Male |  | 79.8 (78.3, 81.2) |  | 64.3 (62.7, 65.8) |  |
| Highest attained education level of head of household, % | 7,269 |  | 9,491 |  | 1100*** |
| No education |  | 32.8 (30.7, 34.9) |  | 16.0 (14.8, 17.3) |  |
| Incomplete or complete primary |  | 24.4 (23.0, 25.9) |  | 18.6 (17.4, 19.8) |  |
| Incomplete or complete secondary |  | 33.1 (31.0, 35.3) |  | 49.4 (47.8, 51.0) |  |
| Education beyond secondary |  | 9.7 (8.7, 10.9) |  | 16.0 (14.8, 17.4) |  |
| Employment status of head of household, % | 7,266 |  | 8,995 |  | 47.8*** |
| Employed |  | 100 |  | 99.3 (99.0, 99.5) |  |
| Unemployed |  | 0 |  | 0.70 (0.51, 0.96) |  |
| Location of household residence, % | 7,273 |  | 9,499 |  | 3700*** |
| Urban |  | 23.2 (21.0, 25.4) |  | 71.6 (69.0, 74.2) |  |
| Rural |  | 76.8 (74.6, 79.0) |  | 28.4 (25.8, 31.0) |  |
| Quintiles of total household expenditure, % in each quintile | 7,273 |  | 9,499 |  | 2500*** |
| Lowest |  | 27.6 (25.3, 30.1) |  | 8.6 (7.6, 9.8) |  |
| Low |  | 24.6 (23.2, 26.1) |  | 11.8 (10.8, 13.0) |  |
| Middle |  | 19.9 (18.5, 21.4) |  | 18.7 (17.3, 20.2) |  |
| High |  | 16.3 (15.0, 17.6) |  | 24.2 (22.8, 25.7) |  |
| Highest |  | 11.6 (10.3, 12.9) |  | 36.6 (34.1, 39.3) |  |
| Total value of household food consumption and expenditures  (per adult equivalent), Ghanaian cedi | 7,273 | 136 (129, 143) | 9,499 | 157 (150, 163) | 7.5*** |
| Total value of household food consumption and expenditures  on ASFs (per adult equivalent), Ghanaian cedi | 7,273 | 34.4 (32.1, 36.6) | 9,493 | 46.6 (44.4, 48.8) | 16.2*** |
| Total value of household food consumption from  own production (per adult equivalent), Ghanaian cedi | 7,273 | 57.4 (52.1, 62.7) | 9,499 | 13.2 (11.2, 15.3) | -25.9*** |
| Total value of household ASF consumption from  own production (per adult equivalent), Ghanaian cedi | 7,273 | 7.3 (5.7, 8.9) | 9,493 | 0.49 (0.24, 0.73) | -16.9*** |
| Ownership of any livestock, % | . |  | . |  | . |
| Yes |  | . |  | . |  |
| No |  | . |  | . |  |
| Ownership of cattle, % | 7,273 |  | . |  | . |
| Yes |  | 11.1 (9.7, 12.6) |  | . |  |
| No |  | 88.9 (87.4, 90.3) |  | . |  |
| Ownership of sheep, % | 7,273 |  | . |  | . |
| Yes |  | 28.7 (26.8, 30.7) |  | . |  |
| No |  | 71.3 (69.3, 73.2) |  | . |  |
| Ownership of goats, % | 7,273 |  | . |  | . |
| Yes |  | 48.0 (45.8, 50.2) |  | . |  |
| No |  | 52.0 (49.8, 54.2) |  | . |  |
| Ownership of pigs, % | 7,273 |  | . |  | . |
| Yes |  | 8.2 (7.0, 9.6) |  | . |  |
| No |  | 91.8 (90.4, 93.0) |  | . |  |
| Ownership of chickens, % | 7,273 |  | . |  | . |
| Yes |  | 82.5 (81.0, 84.0) |  | . |  |
| No |  | 17.5 (16.0, 19.0) |  | . |  |
| Household participation in wild fish capture, % | 7,273 |  | . |  | . |
| Yes |  | 3.1 (2.1, 4.5) |  | . |  |
| No |  | 96.9 (95.5, 97.9) |  | . |  |
| Household participation in fish farming, % | 7,273 |  |  |  |  |
| Yes |  | 0.16 (0.08, 0.34) |  |  |  |
| No |  | 99.8 (99.7, 99.9) |  |  |  |

^a^Values are proportions or means (95% CI) adjusted for the multistage sampling frame of the Ghana Living Standards Survey Round 6 through the use of Taylor-linearized standard errors; ^b^Sample sizes shown are nominal sample sizes; ^c^The two-sided Student’s t-statistics and Pearson’s chi-squared test statistics shown test for differences in means and proportions, respectively, for characteristics among households with livestock and with no livestock; ^d^**P*<0.05; ***P*<0.01; ****P*<0.001.

**Supplemental Table 2.** Sociodemographic characteristics, and health and nutrition indicators among non-pregnant women aged 15-49 years and children aged 6-59 months from the 2014 Ghana Demographic and Health Survey, by household livestock ownership.

|  | Households with any livestock | | Households with no livestock | |  |
| --- | --- | --- | --- | --- | --- |
|  | n | % or mean  (95% CI) | n | % or mean  (95% CI) | t-statistic or Χ^2^ |
| Number of women | 2,152 |  | 2,289 |  |  |
| Number of households | 1,605 |  | 1,875 |  |  |
|  |  |  |  |  |  |
| Sociodemographic characteristics |  |  |  |  |  |
| Household size | 2,152 | 6.0 (5.8, 6.3) | 2,289 | 4.3 (4.1, 4.4) | -23.4*** |
| Age, years | 2,152 | 30.0 (29.5, 30.4) | 2,289 | 29.9 (29.4, 30.2) | 0.31 |
| Sex of head of household, % | 2,152 |  | 2,289 |  | 285*** |
| Female |  | 26.8 (23.7, 30.2) |  | 47.5 (44.7, 50.3) |  |
| Male |  | 73.2 (69.8, 76.3) |  | 52.5 (49.7, 55.3) |  |
| Highest attained education level of woman, % | 2,152 |  | 2,289 |  | 281*** |
| No education |  | 28.8 (25.5, 32.3) |  | 12.8 (11.0, 14.8) |  |
| Incomplete or complete primary |  | 20.5 (18.4, 22.9) |  | 17.0 (15.0, 19.1) |  |
| Incomplete or complete secondary |  | 49.0 (45.7, 52.3) |  | 61.8 (59.2, 64.4) |  |
| Education beyond secondary |  | 1.7 (1.1, 2.6) |  | 8.5 (7.0, 10.1) |  |
| Location of household residence, % | 2,152 |  | 2,289 |  | 709*** |
| Urban |  | 28.7 (24.4, 33.5) |  | 71.2 (66.1, 75.8) |  |
| Rural |  | 71.3 (66.5, 75.6) |  | 28.8 (24.2, 33.9) |  |
| Wealth quintiles, % in each quintile | 2,151 |  | 2,289 |  | 475*** |
| Lowest |  | 19.3 (16.3, 22.7) |  | 6.9 (5.6, 8.6) |  |
| Low |  | 25.8 (23.2, 28.7) |  | 13.5 (11.6, 15.7) |  |
| Middle |  | 22.0 (19.3, 25.1) |  | 18.7 (16.5, 21.0) |  |
| High |  | 16.3 (14.0, 18.9) |  | 22.3 (19.9, 25.0) |  |
| Highest |  | 16.5 (14.0, 19.2) |  | 38.6 (34.9, 42.3) |  |
| Household access to improved water source, % | 2,152 |  | 2,289 |  | 39.7*** |
| Yes |  | 70.1 (66.2, 73.7) |  | 60.0 (56.0, 63.8) |  |
| No |  | 29.9 (26.3, 33.8) |  | 40.0 (36.2, 44.0) |  |
| Household access to improved sanitation source, % | 2,151 |  | 2,289 |  | 5.0* |
| Yes |  | 12.2 (10.2, 14.6) |  | 16.0 (13.3, 19.1) |  |
| No |  | 87.8 (85.4, 89.8) |  | 84.0 (80.9, 86.7) |  |
| Recent anti-malaria indoor residual spraying of household, % | 2,150 |  | 2,258 |  | 157*** |
| Yes |  | 18.8 (14.9, 23.5) |  | 7.8 (5.8, 10.5) |  |
| No |  | 81.2 (76.5, 85.1) |  | 92.2 (89.5, 94.2) |  |
| Household access to mosquito bed net for sleeping, % | 2,152 |  | 2,289 |  | 107*** |
| Yes |  | 83.7 (81.1, 86.0) |  | 68.2 (65.4, 70.9) |  |
| No |  | 16.3 (14.0, 18.8) |  | 31.8 (29.1, 34.6) |  |
| Ownership of any livestock, % | . |  | . |  | . |
| Yes |  | . |  | . |  |
| No |  | . |  | . |  |
| Ownership of non-dairy cattle, % | 2,152 |  | . |  | . |
| Yes |  | 9.6 (7.4, 12.3) |  | . |  |
| No |  | 90.4 (87.7, 92.6) |  | . |  |
| Ownership of dairy cattle, % | 2,152 |  | . |  | . |
| Yes |  | 6.8 (5.0, 9.1) |  | . |  |
| No |  | 93.2 (90.9, 95.0) |  | . |  |
| Ownership of sheep, % | 2,152 |  | . |  | . |
| Yes |  | 26.5 (23.0, 30.2) |  | . |  |
| No |  | 73.5 (69.8, 77.0) |  | . |  |
| Ownership of goats, % | 2,152 |  | . |  | . |
| Yes |  | 48.3 (44.7, 51.8) |  | . |  |
| No |  | 51.7 (48.2, 55.3) |  | . |  |
| Ownership of pigs, % | 2,152 |  | . |  | . |
| Yes |  | 7.6 (6.1, 9.3) |  | . |  |
| No |  | 92.4 (90.7, 93.9) |  | . |  |
| Ownership of chickens, % | 2,150 |  | . |  | . |
| Yes |  | 86.8 (84.4, 88.9) |  | . |  |
| No |  | 13.2 (11.1, 15.6) |  | . |  |
| Ownership of chickens (and no other livestock), % | 2,152 |  | . |  | . |
| Yes |  | 38.3 (34.6, 42.2) |  | . |  |
| No |  | 61.7 (57.8, 65.4) |  | . |  |
|  |  |  |  |  |  |
| Health and nutrition indicators |  |  |  |  |  |
| Anemia status, % | 2,152 |  | 2,289 |  | 5.9 |
| Not anemic |  | 56.6 (53.9, 59.1) |  | 58.5 (55.9, 61.1) |  |
| Mild |  | 34.7 (32.3, 37.2) |  | 32.3 (30.2, 34.6) |  |
| Moderate |  | 8.3 (7.0, 9.8) |  | 8.8 (7.4, 10.5) |  |
| Severe |  | 0.47 (0.22, 0.97) |  | 0.34 (0.14, 0.83) |  |
| Parity | 2,152 | 2.8 (2.7, 2.9) | 2,289 | 2.5 (2.4, 2.7) | -2.2* |
| Current tobacco use | 2,152 |  | 2,287 |  | 0.18 |
| Yes |  | 0.47 (0.11, 1.9) |  | 0.20 (0.07, 0.60) |  |
| No |  | 99.5 (98.1, 99.9) |  | 99.8 (99.4, 99.9) |  |
| Weight status, kg | 2,146 | 59.5 (58.8, 60.3) | 2,283 | 65.3 (64.4, 66.2) | 13.5*** |
|  |  |  |  |  |  |
| Number of children | 1,479 |  | 1,257 |  |  |
| Number of households | 1,092 |  | 993 |  |  |
|  |  |  |  |  |  |
| Sociodemographic characteristics |  |  |  |  |  |
| Household size | 1,478 | 6.5 (6.2, 6.7) | 1,257 | 5.0 (4.8, 5.1) | -15.9*** |
| Age, months | 1,478 | 33.3 (32.4, 34.2) | 1,257 | 31.9 (30.8, 33.1) | -2.3* |
| Sex of child, % | 1,285 |  | 1,102 |  | 3.6 |
| Female |  | 49.8 (46.9, 52.7) |  | 44.7 (41.2, 48.3) |  |
| Male |  | 50.2 (47.3, 53.1) |  | 55.3 (51.7, 58.8) |  |
| Sex of head of household, % | 1,478 |  | 1,257 |  | 110*** |
| Female |  | 19.5 (16.5, 22.9) |  | 33.1 (29.5, 36.9) |  |
| Male |  | 80.5 (77.1, 83.5) |  | 66.9 (63.1, 70.5) |  |
| Highest attained education level of mother, % | 1,332 |  | 1,144 |  | 216*** |
| No education |  | 42.5 (37.6, 47.6) |  | 18.3 (15.3, 21.7) |  |
| Incomplete or complete primary |  | 19.7 (16.9, 22.8) |  | 19.6 (16.6, 23.1) |  |
| Incomplete or complete secondary |  | 37.4 (32.8, 42.2) |  | 56.4 (52.4, 60.4) |  |
| Education beyond secondary |  | 0.40 (0.18, 0.88) |  | 5.7 (4.0, 7.9) |  |
| Location of household residence, % | 1,478 |  | 1,257 |  | 430*** |
| Urban |  | 24.4 (20.3, 29.1) |  | 64.7 (58.9, 70.2) |  |
| Rural |  | 75.6 (70.9, 79.7) |  | 35.3 (29.8, 41.1) |  |
| Wealth quintiles, % in each quintile | 1,476 |  | 1,257 |  | 312*** |
| Lowest |  | 24.8 (21.1, 28.8) |  | 8.7 (7.0, 10.8) |  |
| Low |  | 26.9 (23.9, 30.1) |  | 14.4 (12.0, 17.2) |  |
| Middle |  | 20.7 (17.7, 24.1) |  | 20.1 (17.0, 23.5) |  |
| High |  | 14.1 (11.7, 17.0) |  | 20.7 (17.7, 23.9) |  |
| Highest |  | 13.5 (10.8, 16.7) |  | 36.2 (31.7, 40.9) |  |
| Household access to improved water source, % | 1,478 |  | 1,257 |  | 4.8* |
| Yes |  | 72.3 (67.9, 76.3) |  | 62.7 (58.2, 67.0) |  |
| No |  | 27.7 (23.7, 32.1) |  | 37.3 (33.0, 41.8) |  |
| Household access to improved sanitation source, % | 1,478 |  | 1,257 |  | 0.53 |
| Yes |  | 9.5 (7.5, 11.9) |  | 12.3 (9.4, 15.8) |  |
| No |  | 90.5 (88.1, 92.5) |  | 87.7 (84.2, 90.6) |  |
| Recent anti-malaria indoor residual spraying of household, % | 1,478 |  | 1,244 |  | 59.1*** |
| Yes |  | 18.9 (14.8, 23.9) |  | 9.4 (6.8, 12.7) |  |
| No |  | 81.1 (76.1, 85.2) |  | 90.6 (87.3, 93.2) |  |
| Household access to mosquito bed net for sleeping, % | 1,478 |  | 1,257 |  | 20.8*** |
| Yes |  | 84.4 (81.4, 87.0) |  | 75.2 (71.6, 78.5) |  |
| No |  | 15.6 (13.0, 18.6) |  | 24.8 (21.5, 28.4) |  |
| Ownership of any livestock, % | . |  | . |  | . |
| Yes |  | . |  | . |  |
| No |  | . |  | . |  |
| Ownership of non-dairy cattle, % | 1,478 |  | . |  | . |
| Yes |  | 11.0 (8.6, 14.0) |  | . |  |
| No |  | 89.0 (86.0, 91.4) |  | . |  |
| Ownership of dairy cattle, % | 1,478 |  | . |  | . |
| Yes |  | 8.0 (5.9, 10.7) |  | . |  |
| No |  | 92.0 (89.3, 94.1) |  | . |  |
| Ownership of sheep, % | 1,478 |  | . |  | . |
| Yes |  | 29.1 (25.3, 33.1) |  | . |  |
| No |  | 70.9 (66.9, 74.7) |  | . |  |
| Ownership of goats, % | 1,478 |  | . |  | . |
| Yes |  | 46.8 (42.9, 50.7) |  | . |  |
| No |  | 53.2 (49.3, 57.1) |  | . |  |
| Ownership of pigs, % | 1,478 |  | . |  | . |
| Yes |  | 6.9 (5.3, 9.0) |  | . |  |
| No |  | 93.1 (91.0, 94.7) |  | . |  |
| Ownership of chickens, % | 1,478 |  | . |  | . |
| Yes |  | 87.4 (84.7, 89.6) |  | . |  |
| No |  | 12.6 (10.4, 15.3) |  | . |  |
| Ownership of chickens (and no other livestock), % | 1,478 |  | . |  | . |
| Yes |  | 38.7 (34.7, 42.8) |  | . |  |
| No |  | 61.3 (57.2, 65.3) |  | . |  |
|  |  |  |  |  |  |
| Health and nutrition indicators |  |  |  |  |  |
| Anemia status, % | 1,478 |  | 1,257 |  | 43.0*** |
| Not anemic |  | 26.9 (23.7, 30.2) |  | 40.3 (36.7, 44.0) |  |
| Mild |  | 27.8 (25.1, 30.7) |  | 25.3 (22.4, 28.5) |  |
| Moderate |  | 42.8 (39.2, 46.6) |  | 32.4 (29.0, 35.9) |  |
| Severe |  | 2.5 (1.7, 3.7) |  | 2.0 (1.2, 3.1) |  |
| Fever in previous two weeks, % | 1,285 |  | 1,102 |  | 0.40 |
| Yes |  | 15.4 (13.0, 18.1) |  | 15.2 (12.5, 18.3) |  |
| No |  | 84.6 (81.9, 87.0) |  | 84.8 (81.7, 87.5) |  |
| Diarrhea in previous two weeks, % | 1,285 |  | 1,102 |  | 4.3* |
| Yes |  | 12.5 (10.3, 15.0) |  | 12.7 (10.5, 15.4) |  |
| No |  | 87.5 (85.0, 89.7) |  | 87.3 (84.6, 89.5) |  |
| Consumption of vitamin A supplement in previous 6 months, % | 1,280 |  | 1,091 |  | 8.6** |
| Yes |  | 59.9 (55.6, 64.0) |  | 65.5 (61.3, 69.5) |  |
| No |  | 40.1 (36.0, 44.4) |  | 34.5 (30.5, 38.7) |  |
| Consumption of iron supplement in previous 7 days, % | 1,281 |  | 1,097 |  | 9.3** |
| Yes |  | 21.2 (18.1, 24.8) |  | 25.0 (21.7, 28.6) |  |
| No |  | 78.8 (75.2, 81.9) |  | 75.0 (71.4, 78.3) |  |
| Treatment for intestinal worms in the last 6 months, % | 1,282 |  | 1,100 |  | 28.0*** |
| Yes |  | 34.4 (30.4, 38.6) |  | 40.7 (36.6, 44.9) |  |
| No |  | 65.6 (61.4, 69.6) |  | 59.3 (55.1, 63.4) |  |
| Breastfeeding status, % | 1,285 |  | 1,101 |  | 9.7** |
| Never breastfed |  | 1.0 (0.55, 2.0) |  | 1.0 (0.46, 2.2) |  |
| Ever breastfed and not currently breastfeeding |  | 66.0 (63.3, 68.7) |  | 69.7 (66.2, 72.9) |  |
| Still breastfeeding |  | 32.9 (30.3, 35.6) |  | 29.3 (26.1, 32.8) |  |
| Minimum Dietary Diversity, % | 759 |  | 613 |  | 10.4** |
| Yes |  | 14.3 (11.2, 18.0) |  | 25.5 (20.0, 31.8) |  |
| No |  | 85.7 (82.0, 88.8) |  | 74.5 (68.2, 80.0) |  |
| Consumption of any animal-source foods in past 24 hours, % | 759 |  | 613 |  | 2.5 |
| Yes |  | 46.7 (41.6, 51.9) |  | 54.2 (47.8, 60.3) |  |
| No |  | 53.3 (48.1, 58.4) |  | 45.8 (39.7, 52.1) |  |
| Consumption of meat in past 24 hours, % | 759 |  | 613 |  | 0.46 |
| Yes |  | 9.9 (7.2, 13.3) |  | 12.8 (9.7, 16.7) |  |
| No |  | 90.1 (86.7, 92.8) |  | 87.2 (83.3, 90.3) |  |
| Consumption of fish in past 24 hours, % | 759 |  | 613 |  | 0.46 |
| Yes |  | 36.4 (31.6, 41.4) |  | 42.9 (36.5, 49.6) |  |
| No |  | 63.6 (58.6, 68.4) |  | 57.1 (50.4, 63.5) |  |
| Consumption of eggs in past 24 hours, % | 759 |  | 613 |  | 21.6*** |
| Yes |  | 11.1 (8.3, 14.6) |  | 21.8 (16.8, 27.7) |  |
| No |  | 88.9 (85.4, 91.7) |  | 78.2 (72.3, 83.2) |  |
| Consumption of dairy in past 24 hours, % | 759 |  | 613 |  | 4.2* |
| Yes |  | 0.73 (0.27, 2.0) |  | 3.3 (1.2, 8.9) |  |
| No |  | 99.3 (98.0, 99.7) |  | 96.7 (91.1, 98.8) |  |
| Consumption of organ meats in past 24 hours, % | 759 |  | 613 |  | 2.8 |
| Yes |  | 2.0 (1.1, 3.5) |  | 2.8 (1.7, 4.6) |  |
| No |  | 98.0 (96.5, 98.9) |  | 97.2 (95.4, 98.3) |  |
| Presence of malaria parasites, % | 1,474 |  | 1,252 |  | 33.8*** |
| Yes |  | 32.9 (29.5, 36.6) |  | 20.9 (18.0, 24.2) |  |
| No |  | 67.1 (63.4, 70.5) |  | 79.1 (75.8, 82.0) |  |

^a^Values are proportions or means (95% CI) adjusted for the multistage sampling frame of the 2014 Ghana Demographic and Health Survey and intra-household clustering through the use of Taylor-linearized standard errors; ^b^Sample sizes shown are nominal sample sizes; ^c^The two-sided Student’s t-statistics and Pearson’s chi-squared test statistics shown test for differences in means and proportions, respectively, for characteristics among households with livestock and with no livestock; ^d^**P*<0.05; ***P*<0.01; ****P*<0.001; ^e^Any anemia, mild, moderate and severe anemia, respectively, were defined as Hb <120; 110-119; 80-109; and <80 g/L for women, and Hb <110; 100-109; 70-99; and <70 g/L for children; ^f^Consumption of “meat” refers to consumption of beef, pork, lamb, goat, chicken or duck; consumption of fish refers to consumption of fresh or dried fish or shellfish; ^g^Minimum Dietary Diversity was defined as consumption of four or more of the following seven food groups in the previous 24 hours: 1) grains, roots and tubers, 2) legumes and nuts, 3) dairy products (milk, yogurt, cheese), 4) flesh foods (meat, fish, poultry, and organ meats), 5) eggs, 6) vitamin A-rich fruits and vegetables, and 7) other fruits and vegetables.

**Supplemental Table 3**. Results of multiple logistic regression analyses for the association of household livestock ownership with anemia among children aged 6-59 months using the sub-sample of children for which dietary data were available in the 2014 Ghana Demographic and Health Survey.

|  | OR (95% CI) |
| --- | --- |
| Household livestock ownership | 1.6* (1.0, 2.4) |
| Household size | 1.0 (0.94, 1.1) |
| Age, months | 0.98* (0.97, 0.99) |
| Sex of head of household |  |
| Male (reference) | . |
| Female | 1.7* (1.1, 2.7) |
| Sex of child |  |
| Male (reference) | . |
| Female | 0.87 (0.61, 1.2) |
| Highest attained education level of mother |  |
| No education (reference) | . |
| Incomplete or complete primary | 0.69 (0.41, 1.2) |
| Incomplete or complete secondary | 0.58* (0.36, 0.94) |
| Education beyond secondary | 0.47 (0.13, 1.8) |
| Location of household residence |  |
| Urban (reference) | . |
| Rural | 1.2 (0.75, 1.9) |
| Wealth quintiles |  |
| Lowest (reference) | . |
| Low | 1.3 (0.72, 2.2) |
| Middle | 0.65 (0.37, 1.1) |
| High | 1.0 (0.53, 2.0) |
| Highest | 0.89 (0.41, 1.9) |
| Household access to improved water source |  |
| No (reference) | . |
| Yes | 1.3 (0.89, 1.9) |
| Household access to improved sanitation source |  |
| No (reference) | . |
| Yes | 0.68 (0.41, 1.1) |
| Recent anti-malaria indoor residual spraying of household |  |
| No (reference) | . |
| Yes | 0.32** (0.14, 0.70) |
| Fever in previous two weeks |  |
| No (reference) | . |
| Yes | 2.3** (1.4, 3.9) |
| Diarrhea in previous two weeks |  |
| No (reference) | . |
| Yes | 1.6 (0.98, 2.7) |
| Breastfeeding status |  |
| Never breastfed (reference) | . |
| Ever breastfed and not currently breastfeeding | 0.65 (0.22, 2.0) |
| Still breastfeeding | 0.87 (0.27, 2.8) |
| Presence of malaria parasites |  |
| No (reference) | . |
| Yes | 2.7*** (1.6, 4.4) |
| Minimum Dietary Diversity |  |
| No (reference) | . |
| Yes | 1.0 (0.64, 1.7) |

^a^Values are odds ratios (95% CI) from multiple logistic regression models of the association of household livestock ownership with anemia status adjusting for all covariates shown as well as regional fixed effects. Standard errors are adjusted for intra-household clustering and the multistage sampling frame of the 2014 Ghana Demographic and Health Survey through the use of Taylor-linearized standard errors; ^b^Anemia is modeled as a dichotomous variable defined among women as hemoglobin < 120 g/L, and among children as hemoglobin < 110 g/L; ^c^n = 1,359; ^4^**P*<0.05; ***P*<0.01; ****P*<0.001.

**Supplemental Table 4**. Results of interaction models of logistic regression analyses examining the association of household livestock ownership with anemia among non-pregnant women aged 15-49 years and children aged 6-59 months, respectively.

|  | Women aged 15-49 years | | | |
| --- | --- | --- | --- | --- |
| Model interacting household livestock ownership with… | Education level of woman | Improved water source | Improved sanitation source | Location of household |
|  | OR (95% CI) | OR (95% CI) | OR (95% CI) | OR (95% CI) |
| Household livestock ownership | 0.92 (0.65, 1.3) | 1.2 (0.92, 1.7) | 1.1 (0.86, 1.3) | 1.1 (0.87, 1.4) |
| Highest attained education level of woman |  |  |  |  |
| No education (reference) | . | . | . | . |
| Incomplete or complete primary | 0.79 (0.56, 1.1) | 0.87 (0.69, 1.1) | 0.87 (0.69, 1.1) | 0.87 (0.69, 1.1) |
| Incomplete or complete secondary | 0.77 (0.57, 1.0) | 0.82 (0.66, 1.0) | 0.81* (0.66, 0.99) | 0.81 (0.66, 1.0) |
| Education beyond secondary | 0.53** (0.33, 0.85) | 0.60* (0.40, 0.89) | 0.57** (0.38, 0.85) | 0.59** (0.40, 0.87) |
| Household access to improved water source |  |  |  |  |
| No (reference) | . | . | . | . |
| Yes | 1.2* (1.0, 1.4) | 1.4** (1.1, 1.7) | 1.2* (1.0, 1.4) | 1.2* (1.0, 1.4) |
| Household access to improved sanitation source |  |  |  |  |
| No (reference) | . | . | . | . |
| Yes | 1.4** (1.1, 1.8) | 1.4** (1.1, 1.8) | 1.6** (1.2, 2.2) | 1.4** (1.1, 1.8) |
| Location of household residence |  |  |  |  |
| Urban (reference) | . | . | . | . |
| Rural | 0.81* (0.68, 0.96) | 0.80* (0.67, 0.95) | 0.80* (0.67, 0.95) | 0.87 (0.69, 1.1) |
| Household livestock ownership*highest attained education level of woman |  |  |  |  |
| Own livestock*incomplete or complete primary | 1.2 (0.76, 1.9) | . | . | . |
| Own livestock*incomplete or complete secondary | 1.1 (0.75, 1.6) | . | . | . |
| Own livestock*education beyond secondary | 1.5 (0.57, 4.1) | . | . | . |
| Household livestock ownership*household access to improved water source |  |  |  |  |
| Own livestock*improved water source | . | 0.75 (0.54, 1.0) | . | . |
| Household livestock ownership*household access to improved sanitation source |  |  |  |  |
| Own livestock*improved sanitation source | . | . | 0.71 (0.47, 1.1) | . |
| Household livestock ownership*location of household residence |  |  |  |  |
| Own livestock*urban location | . | . | . | 0.83 (0.59, 1.2) |
|  |  |  |  |  |
|  | Children aged 6-59 months | | | |
| Model interacting household livestock ownership with… | Education level of mother | Improved water source | Improved sanitation source | Location of household |
|  | OR (95% CI) | OR (95% CI) | OR (95% CI) | OR (95% CI) |
| Household livestock ownership | 1.3 (0.82, 2.1) | 1.5 (0.92, 2.4) | 1.5* (1.1, 2.0) | 1.4 (0.95, 2.2) |
| Highest attained education level of mother |  |  |  |  |
| No education (reference) | . | . | . | . |
| Incomplete or complete primary | 0.69 (0.39, 1.2) | 0.71* (0.50, 0.99) | 0.71* (0.50, 0.99) | 0.70* (0.50, 0.99) |
| Incomplete or complete secondary | 0.55* (0.35, 0.87) | 0.60** (0.44, 0.83) | 0.60** (0.44, 0.83) | 0.60** (0.44, 0.83) |
| Education beyond secondary | 0.42 (0.17, 1.0) | 0.42* (0.18, 0.99) | 0.42 (0.17, 1.0) | 0.42* (0.18, 0.99) |
| Household access to improved water source |  |  |  |  |
| No (reference) | . | . | . | . |
| Yes | 1.1 (0.85, 1.4) | 1.1 (0.79, 1.6) | 1.1 (0.86, 1.5) | 1.1 (0.85, 1.5) |
| Household access to improved sanitation source |  |  |  |  |
| No (reference) | . | . | . | . |
| Yes | 0.61* (0.42, 0.89) | 0.61* (0.42, 0.89) | 0.60* (0.36, 0.99) | 0.62* (0.43, 0.89) |
| Location of household residence |  |  |  |  |
| Urban (reference) | . | . | . | . |
| Rural | 0.94 (0.68, 1.3) | 0.95 (0.69, 1.3) | 0.95 (0.69, 1.3) | 0.94 (0.65, 1.4) |
| Household livestock ownership*highest attained education level of mother |  |  |  |  |
| Own livestock*incomplete or complete primary | 1.0 (0.53, 2.0) | . | . | . |
| Own livestock*incomplete or complete secondary | 1.2 (0.70, 2.2) | . | . | . |
| Own livestock*education beyond secondary | 0.30 (0.05, 1.9) | . | . | . |
| Household livestock ownership*household access to improved water source |  |  |  |  |
| Own livestock*improved water source | . | 0.97 (0.59, 1.6) | . | . |
| Household livestock ownership*household access to improved sanitation source |  |  |  |  |
| Own livestock*improved sanitation source | . | . | 1.1 (0.54, 2.1) | . |
| Household livestock ownership*location of household residence |  |  |  |  |
| Own livestock*urban location | . | . | . | 1.0 (0.59, 1.8) |

^a^Values are odds ratios (95% CI) from multiple logistic regression models of the association of household livestock ownership with anemia status that control for all covariates shown, household size, sex of household head, location of household residence, wealth quintiles, recent anti-malaria indoor residual spraying of household, household access to mosquito bed net for sleeping, and regional fixed effects. Among women, the following covariates are also adjusted for: age of woman, current tobacco use, weight status, and parity. Among children, the following covariates are also adjusted for: age of child, sex of child, fever in previous two weeks, diarrhea in previous two weeks, consumption of vitamin A supplement in previous 6 months, consumption of iron supplement in previous 7 days, treatment for intestinal worms in the last 6 months, breastfeeding status, and presence of malaria parasites. Standard errors are adjusted for intra-household clustering and the multistage sampling frame of the 2014 Ghana Demographic and Health Survey through the use of Taylor-linearized standard errors; ^b^Anemia is modeled as a dichotomous variable defined among women as hemoglobin < 120 g/L, and among children as hemoglobin < 110 g/L; ^c^Sample sizes: Women: n = 4,392; children: n = 2,336; ^d^**P*<0.05; ***P*<0.01; ****P*<0.001.

**Supplemental Table 5**. Monetary value per adult equivalent of household food consumption and expenditures in the previous 35 days among participants of the Ghana Living Standards Survey Round 6, by own produced food and purchased food.

|  |  | Total sample |  | Households with livestock |  | Households with no livestock |  |
| --- | --- | --- | --- | --- | --- | --- | --- |
|  | n | Mean  (95% CI) | n | Mean  (95% CI) | n | Mean  (95% CI) | t-statistic |
|  |  |  |  |  |  |  |  |
| Value of household food consumption (own produced food) |  |  |  |  |  |  |  |
| All food consumption per adult equivalent | 16,772 | 28.0 (25.3, 30.7) | 7,273 | 57.4 (52.1, 62.7) | 9,499 | 13.2 (11.2, 15.3) | -25.9*** |
| ASF consumption per adult equivalent | 16,766 | 2.8 (2.2, 3.4) | 7,273 | 7.3 (5.7, 8.9) | 9.493 | 0.49 (0.24, 0.73) | -16.9*** |
| Meat consumption per adult equivalent | 16,766 | 0.73 (0.30, 1.2) | 7,273 | 2.0 (0.73, 3.3) | 9,493 | 0.09 (0.05, 0.13) | -6.9*** |
| Chicken consumption per adult equivalent | 16,766 | 1.1 (0.91, 1.3) | 7,273 | 3.0 (2.6, 3.4) | 9,493 | 0.12 (0.02, 0.25) | -25.2*** |
| Fish consumption per adult equivalent | 16,766 | 0.64 (0.33, 0.95) | 7,273 | 1.5 (0.69, 2.3) | 9,493 | 0.21 (0.06, 0.35) | -5.5*** |
| Milk consumption per adult equivalent | 16,766 | 0.01 (0.00, 0.01) | 7,273 | 0.02 (0.01, 0.03) | 9,493 | 0.00 (0.00, 0.00) | -4.5*** |
| Egg consumption per adult equivalent | 16,766 | 0.17 (0.11, 0.22) | 7,273 | 0.38 (0.32, 0.45) | 9,493 | 0.06 (0.00, 0.13) | -6.1*** |
|  |  |  |  |  |  |  |  |
| Value of household food expenditures (purchased food) |  |  |  |  |  |  |  |
| All food expenditures per adult equivalent | 16,772 | 122 (117, 127) | 7,273 | 78.9 (75.1, 82.6) | 9,499 | 143 (137, 150) | 30.6*** |
| ASF expenditures per adult equivalent | 16,772 | 39.7 (38.0, 41.4) | 7,273 | 27.1 (25.4, 28.7) | 9,499 | 46.1 (44.0, 48.3) | 29.8*** |
| Meat expenditures per adult equivalent | 16,772 | 6.6 (6.1, 7.1) | 7,273 | 4.6 (3.8, 5.4) | 9,499 | 7.6 (7.0, 8.2) | 12.8*** |
| Chicken expenditures per adult equivalent | 16,772 | 3.9 (3.6, 4.2) | 7.273 | 2.3 (2.0, 2.5) | 9.499 | 4.7 (4.3, 5.1) | 18.7*** |
| Fish expenditures per adult equivalent | 16,772 | 24.1 (23.0, 25.3) | 7,273 | 17.9 (16.8, 19.1) | 9,499 | 27.3 (25.8, 28.7) | 23.3*** |
| Milk expenditures per adult equivalent | 16,772 | 3.1 (2.9, 3.3) | 7,273 | 1.4 (1.2, 1.5) | 9,499 | 3.9 (3.7, 4.2) | 29.3*** |
| Egg expenditures per adult equivalent | 16,772 | 1.9 (1.8, 2.0) | 7,273 | 0.73 (0.66, 0.81) | 9,499 | 2.5 (2.3, 2.7) | 30.7*** |

^a^Values are means (95% CI) in units of Ghanaian cedi adjusted for the multistage sampling frame of the Ghana Living Standards Survey Round 6 through the use of Taylor-linearized standard errors; ^b^Sample sizes shown are nominal sample sizes; ^c^The two-sided Student’s t-statistics shown test for differences in means for characteristics among households with livestock and with no livestock. ^d^**P*<0.05; ***P*<0.01; ****P*<0.001; ^e^Own produced “meat” includes: beef, mutton, pork, goat, other domestic meat, and wild game; purchased “meat” includes: corned beef, pork, beef, goat, mutton, bushmeat or wild game, and other meat; purchased “fish” includes: crustaceans, fish (fresh, frozen, dried, canned, fried, smoked, salted); purchased “milk” includes: fresh, powdered, or tinned milk.

**Supplemental Table 6**. Covariate results from multiple linear regression analyses for the association of household ownership of livestock with the monetary value per adult equivalent of household food consumption and expenditures in the previous 35 days, by food type, among participants of the Ghana Living Standards Survey Round 6.

|  | Value of household food consumption (own produced food) and expenditures (purchased food) per adult equivalent | | | |
| --- | --- | --- | --- | --- |
|  | All food | ASFs | Chicken | Eggs |
|  | Coeff. (95% CI) | Coeff. (95% CI) | Coeff. (95% CI) | Coeff. (95% CI) |
| Main independent variable: Household ownership of any livestock |  |  |  |  |
| Sex of head of household |  |  |  |  |
| Male (reference) | . | . | . | . |
| Female | 18.4*** (11.0, 25.8) | 6.4*** (4.2, 8.7) | 0.31 (-0.26, 0.88) | 0.48*** (0.24, 0.72) |
| Age of head of household (years) | 0.30* (0.07, 0.53) | 0.10** (0.03, 0.17) | -0.00 (-0.02, 0.01) | -0.02*** (-0.02, -0.01) |
| Education of head of household |  |  |  |  |
| No education (reference) | . | . | . | . |
| Incomplete or complete primary | -3.1 (-12.3, 6.2) | -0.03 (-2.2, 2.1) | 0.08 (-0.57, 0.73) | -0.03 (-0.24, 0.18) |
| Incomplete or complete secondary | -3.2 (-13.7, 7.2) | -0.15 (-2.6, 2.3) | -0.52 (-1.2, 0.12) | 0.05 (-0.13, 0.24) |
| Education beyond secondary | 18.8** (6.2, 31.5) | 9.3*** (5.6, 13.0) | 0.70 (-0.12, 1.5) | 0.54* (0.13, 0.94) |
| Household size | -10.3*** (-11.8, -8.7) | -2.6*** (-3.0, -2.2) | -0.25*** (-0.34, -0.15) | -0.13*** (-0.17, -0.10) |
| Quintiles of total household expenditure |  |  |  |  |
| Lowest (reference) | . | . | . | . |
| Low | 24.5*** (16.6, 32.5) | 6.9*** (5.1, 8.7) | 1.5*** (1.0, 2.0) | 0.14 (-0.05, 0.33) |
| Middle | 45.5*** (34.8, 56.2) | 13.5*** (11.2, 15.7) | 2.3*** (1.7, 3.0) | 0.47*** (0.22, 0.72) |
| High | 69.6*** (57.4, 81.7) | 22.6*** (19.7, 25.5) | 3.8*** (3.1, 4.5) | 0.96*** (0.66, 1.3) |
| Highest | 119*** (106, 132) | 39.8*** (36.3, 43.3) | 5.9*** (5.0, 6.8) | 2.5*** (2.1, 2.8) |
| Location of household residence |  |  |  |  |
| Urban (reference) | . | . | . | . |
| Rural | 18.4** (7.9, 28.8) | 2.0 (-0.97, 4.9) | 1.6*** (0.94, 2.3) | -0.15 (-0.36, 0.07) |
|  |  |  |  |  |
| Main independent variable: Household ownership of chickens |  |  |  |  |
| Sex of head of household | . | . |  |  |
| Male (reference) | . | . | . | . |
| Female | . | . | 0.33 (-0.23, 0.90) | 0.49*** (0.25, 0.73) |
| Age of head of household (years) | . | . | -0.00 (-0.02, 0.01) | -0.02*** (-0.02, -0.01) |
| Education of head of household | . | . |  |  |
| No education (reference) | . | . | . | . |
| Incomplete or complete primary | . | . | 0.06 (-0.59, 0.71) | -0.03 (-0.24, 0.17) |
| Incomplete or complete secondary | . | . | -0.52 (-1.2, 0.12) | 0.05 (-0.13, 0.24) |
| Education beyond secondary | . | . | 0.69 (-0.13, 1.5) | 0.54** (0.13, 0.94) |
| Household size | . | . | -0.25*** (-0.35, -0.16) | -0.14*** (-0.17, -0.10) |
| Quintiles of total household expenditure | . | . |  |  |
| Lowest (reference) | . | . | . | . |
| Low | . | . | 1.5*** (1.0, 2.0) | 0.14 (-0.05, 0.33) |
| Middle | . | . | 2.4*** (1.7, 3.0) | 0.47*** (0.22, 0.73) |
| High | . | . | 3.8*** (3.1, 4.6) | 0.96*** (0.67, 1.3) |
| Highest | . | . | 6.0*** (5.1, 6.9) | 2.5*** (2.1, 2.8) |
| Location of household residence | . | . |  |  |
| Urban (reference) | . | . | . | . |
| Rural | . | . | 1.5*** (0.85, 2.2) | -0.18 (-0.39, 0.03) |
|  |  |  |  |  |
|  | Value of household food consumption (own produced food) per adult equivalent | | | |
|  | All food | ASFs | Chicken | Eggs |
|  | Coeff. (95% CI) | Coeff. (95% CI) | Coeff. (95% CI) | Coeff. (95% CI) |
| Main independent variable: Household ownership of any livestock | 31.2***  (25.8, 36.5) | 5.7***  (4.7, 6.8) | 2.7***  (2.2, 3.2) | 0.39***  (0.30, 0.48) |
| Sex of head of household |  |  |  |  |
| Male (reference) | . | . | . | . |
| Female | -5.1** (-8.5, -1.8) | -1.6*** (-2.3, -0.95) | -0.63*** (-0.97, -0.29) | 0.08 (-0.10, 0.25) |
| Age of head of household (years) | 0.31*** (0.17, 0.45) | 0.01 (-0.03, 0.06) | 0.00 (-0.01, 0.01) | -0.00 (-0.00, 0.00) |
| Education of head of household |  |  |  |  |
| No education (reference) | . | . | . | . |
| Incomplete or complete primary | 0.05 (-4.7, 4.8) | -0.33 (-1.5, 0.80) | -0.05 (-0.54, 0.43) | -0.06 (-0.17, 0.05) |
| Incomplete or complete secondary | -1.8 (-7.4, 3.8) | -2.3*** (-3.5, -1.1) | -0.63** (-1.0, -0.20) | -0.11* (-0.21, -0.01) |
| Education beyond secondary | -3.6 (-10.0, 2.8) | -1.4 (-3.5, 0.74) | -0.60* (-1.1, -0.12) | 0.06 (-0.25, 0.36) |
| Household size | -2.4*** (-3.2, -1.5) | -0.10 (-0.25, 0.05) | -0.11** (-0.17, -0.04) | -0.02* (-0.04, -0.00) |
| Quintiles of total household expenditure |  |  |  |  |
| Lowest (reference) | . | . | . | . |
| Low | 6.4 (-0.90, 13.8) | 1.7* (0.28, 3.1) | 0.85*** (0.46, 1.2) | -0.01 (-0.18, 0.16) |
| Middle | 7.7 (-1.2, 16.6) | 2.0** (0.58, 3.5) | 0.98*** (0.53, 1.4) | 0.02 (-0.18, 0.21) |
| High | 5.3 (-4.0, 14.7) | 2.7** (0.90, 4.4) | 1.1*** (0.65, 1.6) | -0.01 (-0.24, 0.22) |
| Highest | 6.1 (-3.8, 16.1) | 3.4*** (1.6, 5.1) | 1.3*** (0.71, 2.0) | 0.05 (-0.22, 0.31) |
| Location of household residence |  |  |  |  |
| Urban (reference) | . | . | . | . |
| Rural | 23.2*** (16.6, 29.9) | 1.3* (0.06, 2.6) | 0.55** (0.20, 0.89) | 0.07 (-0.01, 0.15) |
|  |  |  |  |  |
| Main independent variable: Household ownership of chickens | . | . | 3.2***  (2.7, 3.7) | 0.46***  (0.37, 0.55) |
| Sex of head of household | . | . |  |  |
| Male (reference) | . | . | . | . |
| Female | . | . | -0.62*** (-0.96, -0.29) | 0.08 (-0.10, 0.25) |
| Age of head of household (years) | . | . | 0.00 (-0.01, 0.01) | -0.00 (-0.00, 0.00) |
| Education of head of household | . | . |  |  |
| No education (reference) | . | . | . | . |
| Incomplete or complete primary | . | . | -0.07 (-0.55, 0.41) | -0.06 (-0.17, 0.05) |
| Incomplete or complete secondary | . | . | -0.64** (-1.1, -0.22) | -0.11* (-0.21, -0.01) |
| Education beyond secondary | . | . | -0.63** (-1.1, -0.16) | 0.05 (-0.25, 0.35) |
| Household size | . | . | -0.11** (-0.17, -0.04) | -0.02* (-0.04, -0.00) |
| Quintiles of total household expenditure | . | . |  |  |
| Lowest (reference) | . | . | . | . |
| Low | . | . | 0.89*** (0.51, 1.3) | -0.01 (-0.18, 0.17) |
| Middle | . | . | 1.0*** (0.57, 1.5) | 0.02 (-0.18, 0.22) |
| High | . | . | 1.2*** (0.72, 1.7) | -0.00 (-0.23, 0.23) |
| Highest | . | . | 1.4*** (0.78, 2.0) | 0.06 (-0.21, 0.32) |
| Location of household residence | . | . |  |  |
| Urban (reference) | . | . | . | . |
| Rural | . | . | 0.50** (0.16, 0.84) | 0.07 (-0.01, 0.14) |

^a^Values are partial regression coefficients (95% CI) from multiple linear regression models of the association of household ownership of livestock with the monetary value per adult equivalent of household food consumption and expenditures, by food type. Each column represents a unique regression model using the variable noted in the column header as the main dependent variable, and the main independent variable shown above the list of covariates. Models are adjusted for the covariates shown as well as for the employment status of household head, and regional fixed effects. Standard errors are adjusted for the multistage sampling frame of the of the Ghana Living Standards Survey Round 6 through the use of Taylor-linearized standard errors. Monetary value are in units of Ghanaian cedi; ^b^Sample size: n = 16,246; ^c^**P*<0.05; ***P*<0.01; ****P*<0.001; ^d^Abbreviations: ASFs: animal-source foods; ^e^Animal-source foods include non-chicken meat, chicken, fish, milk and eggs.

**Supplemental Table 7**. Direct, indirect, and total effects of household livestock ownership on child anemia assessing mediation by recent consumption of animal-source foods.

|  | Mediating animal-source food (ASF) variable | | | |
| --- | --- | --- | --- | --- |
|  | Any ASF | Meat | Dairy | Organ meat |
|  | Coefficient (SE) | Coefficient (SE) | Coefficient (SE) | Coefficient (SE) |
| Direct effect | 0.097*** (0.024) | 0.097*** (0.024) | 0.096*** (0.024) | 0.094*** (0.024) |
| Indirect effect | -0.002 (0.001) | -0.001 (0.001) | 0.0002 (0.001) | 0.002 (0.002) |
| Total effect | 0.096*** (0.024) | 0.096*** (0.024) | 0.096*** (0.024) | 0.096*** (0.024) |

^a^Coefficients are standardized path coefficients from models using maximum-likelihood estimation with robust standard errors. The coefficient for the direct effect is the partial regression coefficient of the association of household livestock ownership with child anemia, controlling for the relevant variable indicating consumption of ASFs. The indirect effect of household livestock ownership on child anemia is calculated as the product of the path coefficient between household livestock ownership and consumption of ASFs, and the path coefficient between consumption of ASFs and child anemia. The total effect is the sum of the direct and indirect effects of livestock ownership on child anemia; ^b^Child anemia is modeled as a dichotomous variable defined as hemoglobin < 110 g/L; ^c^n = 1,373; ^d^**P*<0.05; ***P*<0.01; ****P*<0.001; eAll dietary variables are calculated from qualitative 24-hour recall data on consumption of select food groups; consumption of “meat” refers to consumption of beef, pork, lamb, goat, chicken or duck; consumption of “any ASF” refers to consumption of meat, fresh or dried fish or shellfish, milk, cheese, eggs, and/or organ meats.
