# Supplementary figures and images for: Livestock ownership is associated with higher odds of anaemia among preschool‐aged children, but not women of reproductive age in Ghana
Source: Matern Child Nutr. 2018 Apr 2;14(3):e12604. doi: 10.1111/mcn.12604 (PMC6055803; doi:10.1111/mcn.12604)

Supplemental Figure 1

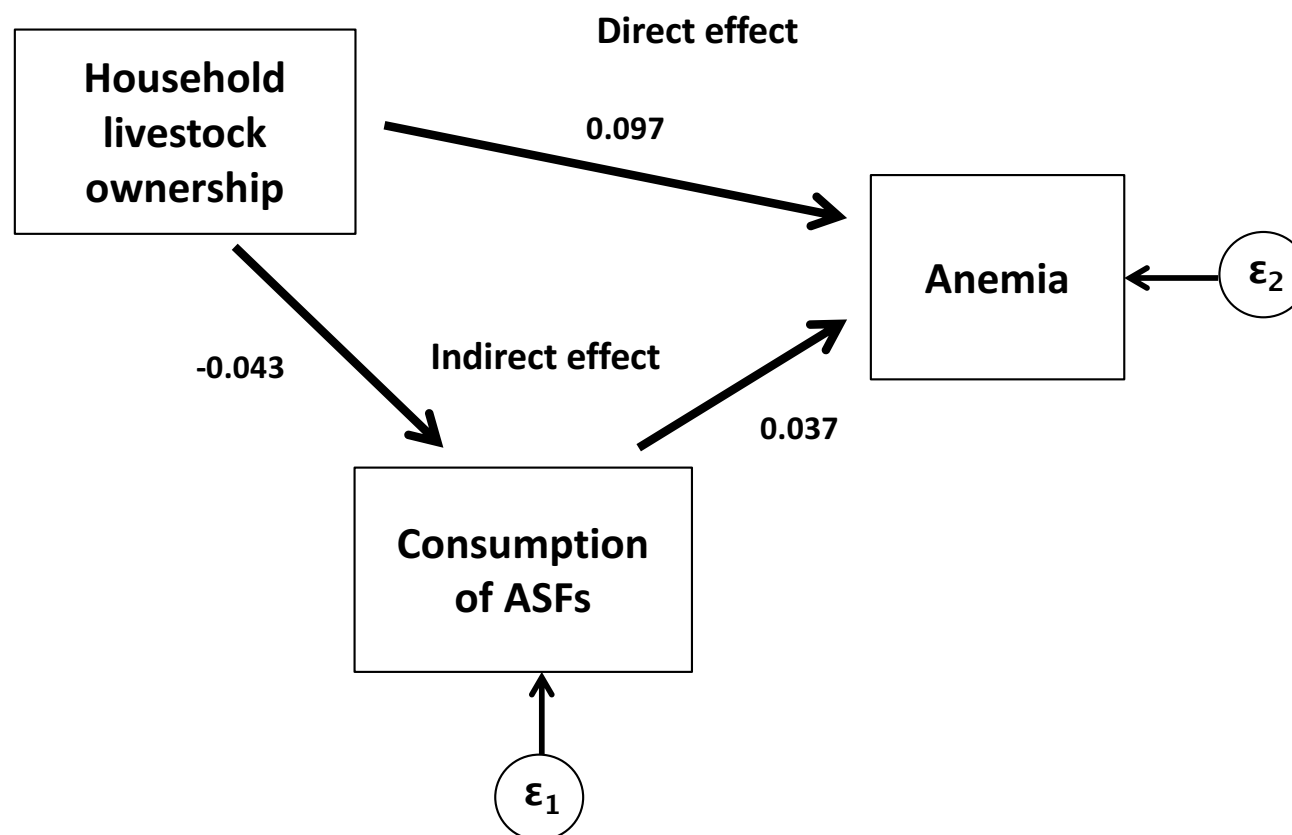

Supplement: Supplementary file 1 — Figure S1. Path diagram of the direct and indirect effects of household livestock ownership on child anemia with mediation by consumption of any animal‐source foods. Table S1. Sociodemographic characteristics of households participating in the Ghana Living Standards Survey Round 6, by household livestock ownership. Table S2. Sociodemographic characteristics, and health and nutrition indicators among non‐pregnant women aged 15–49 years and children aged 6–59 months from the 2014 Ghana Demographic and Health Survey, by household livestock ownership. Table S3. Results of multiple logistic regression analyses for the association of household livestock ownership with anemia among children aged 6–59 months using the sub‐sample of children for which dietary data were available in the 2014 Ghana Demographic and Health Survey. Table S4. Results of interaction models of logistic regression analyses examining the association of household livestock ownership with anemia among non‐pregnant women aged 15–49 years and children aged 6–59 months, respectively. Table S5. Monetary value per adult equivalent of household food consumption and expenditures in the previous 35 days among participants of the Ghana Living Standards Survey Round 6, by own produced food and purchased food. Table S6. Covariate results from multiple linear regression analyses for the association of household ownership of livestock with the monetary value per adult equivalent of household food consumption and expenditures in the previous 35 days, by food type, among participants of the Ghana Living Standards Survey Round 6. Table S7. Direct, indirect, and total effects of household livestock ownership on child anemia assessing mediation by recent consumption of animal‐source foods. [file MCN-14-e12604-s001.docx › MCN_12604-Sup-0002-SupplementalFigure1.pdf]
